# Care quality following intrauterine death in Spanish hospitals: results from an online survey

**DOI:** 10.1186/s12884-017-1630-z

**Published:** 2018-01-10

**Authors:** Paul Richard Cassidy

**Affiliations:** 1 Universidad Complutense de Madrid, Facultad de Ciencia Política y Sociología, Somosaguas, Pozuelo de Alarcon, 28223 Madrid, Spain; 2 Umamanita (Stillbirth Charity), C/ Hierbabuena 15, Esc B, 4 Izq, 28039 Madrid, Spain

**Keywords:** Stillbirth, Late miscarriage, Termination of pregnancy, Perinatal bereavement, Hospital care, Mode of delivery, Sedatives, Perinatal autopsy, Postmortem contact, Linking objects

## Abstract

**Background:**

The objective of the study was to evaluate practices in Spanish hospitals after intrauterine death in terms of medical/ technical care and bereavement support care.

**Methods:**

A cross-sectional descriptive study using an online self-completion questionnaire. The population was defined as women who had experienced an intrauterine fetal death between sixteen weeks and birth, either through spontaneous late miscarriage/stillbirth or termination of pregnancy for medical reasons. Respondents were recruited through an online advertisement on a stillbirth charity website and social media. The analysis used Pearson’s chi-squared (*p* ≤ 0.05) test of independence to cross-analyse for associations between objective measures of care quality and independent variables.

**Results:**

Responses from 796 women were analysed. Half of the women (52.9%) had postmortem contact with their baby. 30.4% left the hospital with a least one linking object or a photograph. In 35.8% of cases parents weren’t given any option to recover the body/remains. 22.9% of births ≥26 weeks gestation were by caesarean, with a significant (*p* < 0.001) difference between public hospitals (16.8%) and private hospitals (41.5%). 29.3% of respondents were not accompanied during the delivery. 48.0% of respondents recalled being administered sedatives at least once during the hospital stay. The autopsy rate in stillbirth cases (≥ 20 weeks) was 70.5% and 44.4% in cases of termination of pregnancy (all gestational ages). Consistent significant (*p* < 0.05) differences in care practices were found based on gestational age and type of hospital (public or private), but not to other variables related to socio-demographics, pregnancy history or details of the loss/death. Intrauterine deaths at earlier gestational ages received poorer quality care.

**Conclusions:**

Supportive healthcare following intrauterine death is important to women’s experiences in the hospital and beneficial to the grief process. Many care practices that are standard in other high-income countries are not routine in Spanish hospitals. Providing such care is a relatively new phenomenon in the Spanish health system, the results provide a quality benchmark and identify a number of areas where hospitals could make improvements to care practices that should have important psychosocial benefits for women and their families.

**Electronic supplementary material:**

The online version of this article (10.1186/s12884-017-1630-z) contains supplementary material, which is available to authorized users.

## Background

Diagnosis of an intrauterine death, or life limiting fetal anomalies, is a devastating experience for many women, their partners and families which can have an enduring impact on their lives [1–3]. Bereaved mothers have a high risk of complicated grief [4, 5] and many experience anxiety, depression and post-traumatic stress disorder [6, 7]. Despite the inherent sadness of their situation, when they receive meaningful support in a caring environment, many women and their partners have positive memories of the birth and their time in the hospital [8, 9].

Over the last few decades, care practices have developed to encompass specific bereavement support actions focused on psychosocial care, which complement medical/ technical aspects of care related to the management of the birth and the death of the baby. The provision of meaningful care stresses the development of an empathetic and trusting relationship between healthcare professionals (HPs) and bereaved women, their partners and other family members [10]. As parents navigate a very difficult few days in the hospital, effective communication and evidence based information are crucial for the many important decisions they have to make about medical care and rituals related to the baby [11, 12]. Despite disagreement about how active a role HPs should take in promoting postmortem contact [13, 14], key aspects of care related to ritual, include: supporting parents to see, hold and interact with their baby, regardless of gestational age or condition; helping them to keep objects related to the baby or birth; and providing options and support for a respectful disposition [1, 15–17].

In terms of medical/technical aspects of care during the hospital stay, this paper deals with three components: the birth, autopsy, and the administration of psychotropic medication. Choosing an appropriate birthing method depends on gestational age, maternal clinical history, medical condition on presentation and the woman’s preferences [12, 18]. In the absence of medical indications to the contrary, the timing of delivery is not critical, and vaginal birth is recommended following either expectant management, induction of labour, or both [12, 18]. As parents seek answers and struggle to come to terms with an unexpected death, a postmortem examination can have emotional benefits and help to allay feelings of guilt [19, 20]. This can also provide useful information for guidance in future pregnancies and make a crucial contribution to the development of medical knowledge about stillbirth etiology [21]. The administration of benzodiazepines during the hospital stay isn’t recommended as a routine intervention as no solid evidence exists of any benefits to the patient in terms of sleep, grief and or trauma [22, 23], although some studies suggest that the practice may be widespread [23–25].

Only very recently has the Spanish national health system recognised the need for specialised bereavement support care following intrauterine death [26]. There is very little data available on care practices, which makes it difficult to assess care quality and benchmark against other countries. Based on a national survey of women who experienced an intrauterine death in the Spanish health system, the data reported in this article focuses on measurable content of care rather than subjective experiences, which will be addressed in future publications. The project was initiated and promoted by the parent support association Umamanita.

## Methods

### Design

A cross-sectional descriptive study was carried out using an online survey.

### Instrument development

The anonymous online self-completion questionnaire was developed following an extensive process, which included: exploratory qualitative interviews (with support association experts, parents and health professionals); content validation with a panel of 14 parents and 10 health professionals; three cognitive interviews with parents on the first draft questionnaire; and, two phases of pilot testing of the online version. The instrument contained 102 questions and assessed a wide-range of objective and subjective aspects of care quality, as well as a series of questions on pregnancy history and socio-demographics (see Additional files 1 and 2 for the Spanish and English translated version of the questionnaire). The mean time to complete the questionnaire was 44:48 min (SD = 22:25 mins).

### Population

The population was defined as women who experienced a spontaneous intrauterine death (late miscarriage/stillbirth) or termination of pregnancy for medical reasons (threat to the mother’s health or fetal anomaly) between 16 weeks gestation and up to and including intrapartum death, within the Spanish healthcare system. Women’s partners were not invited to participate in the study.

### Fieldwork and sample

Fieldwork took place between June 2013 and June 2016. Respondents were recruited through a convenience sampling technique (snowballing), including advertisements on support associations’ websites. Following data purification, 796 women, whose baby had died within the previous 60 months, were deemed to have validly completed the questionnaire. Data purification involved screening out respondents from outside the Spanish national territory (E.g. Latin America), early neonatal deaths and insufficiently complete questionnaires (see Additional file 3 for more details on sampling, data purification and the characteristics of abandoned interviews). The mean age of respondents at the time of the death was 33.7 (SD = 4.30) years and an average of 13.76 (SD = 15.3) months had passed between the death/loss and completion of the survey. See Table 1 for more details on the characteristics of the sample.

**Table 1:** Characteristics of the sample

Sample	796
Socio-demographic details	*n* (%)
Age at the time of the loss (years)	
< 30	125 (17.0)
30–34	364 (45.7)
35–39	228 (28.6)
≥ 40	69 (8.7)
Education	
Lower second level or less	144 (18.1)
Upper second level	183 (23.0)
Third level	496 (58.9)
Nationality	
Spanish	755 (94.8)
Foreign resident	41 (5.2)
Occupation	
Professional, technical or management	348 (43.7)
Administration, commercial activity	237 (29.8)
Services or manual worker	96 (12.1)
Homemaker	67 (8.4)
Other	42 (5.3)
Not stated	6 (0.8)
Marital status at the time of the loss/death	
Married, civil union or cohabiting	769 (96.6)
Single	22 (2.8)
Separated or divorced	4 (0.5)
Widowed	1 (0.1)
Habitat	
Urban	588 (73.9)
Rural	207 (26.0)
Not stated	1 (0.1)
Previous contact with a support association	
Yes (email, support groups, events)	321 (40.4)
No (website only or none)	470 (59.0)
Not stated	5 (0.6)
Pregnancy history	
Primigravida	
Yes	426 (53.5)
No	370 (46.5)
Living children before the loss	
Yes	253 (31.8)
No	543 (68.2)
Previous pregnancy loss or perinatal death	
Yes	197 (24.7)
No	596 (74.9)
Not stated	3 (0.4)
Details of the pregnancy and loss/death	*n* (%)
Gestational age (in weeks)	
16–19	128 (16.1)
20–25	193 (24.2)
26–33	151 (19.0)
≥ 34	324 (40.7)
Type of death	
Spontaneous intrauterine death	605 (76.0)
(Includes death during labour)	21
Termination for medical reasons	189 (23.7)
Not stated	2 (0.3)
Type of pregnancy	
Singular	740 (93.0)
Multiple no survivors	24 (3.0)
Multiple with survivor(s)	32 (4.0)
Time between the death and completing survey	
≤ 3 months	299 (37.6)
4 to 12 months	203 (25.5)
13 to 24 months	119 (14.9)
25 to 60 months	175 (22.0)
Type of hospital	
Public	593 (74.5)
Private	203 (25.5)

### Analysis

The sample data is un-weighted. The analysis used Pearson’s Chi-squared test of independence to cross-analyse for associations between objective measures of care quality and independent variables, including: details of the death (gestational age, type of death, type of pregnancy, time since the death); pregnancy history (% primagravia, living children before the death, previous pregnancy loss or perinatal death); socio-demographics (age, marital status, educational attainment, occupation, nationality, urban/rural habitat); and type of hospital (public/private). Statistical significance was set at *p* < 0.05 and the strength of association between variables was measured by calculating effect size using the *phi-correlation* coefficient for 2 × 2 tables and *Cramér’s V* for larger tables. Effect size was evaluated according to the levels set out in Gravetter and Wallnau [27]. Only gestational age and type of hospital were found to have consistently significant relationships with the dependent variables under measure. The article presents frequency data for each dependent variable and 2 × 4 (gestational age in 4 categories) and 2 × 2 (class of hospital in 2 categories) categorical cross-tabulation tables, which include Chi-squared and effect size values. Missing data values for each dependent variable are identified in the data tables as ‘Not stated’. Further details on the project background, questionnaire design process and methodology are available in Additional file 3.

## Results

### Postmortem contact, linking objects and photographs

Respondents were asked if they or their partner had seen, touched or held the baby. The rate of visual postmortem contact was 52.9% for mothers and 58.9% for their partners. The percentage of mothers who saw their baby was significantly associated (*p* < 0.001) with gestational age, with a large effect size. The proportion of women who had visual contact rose according to the advancement of the pregnancy: in cases from 16 to 19 weeks, 28.9% of mothers saw their baby, compared to 74.1% ≥ 34 weeks (see Table 2).

**Table 2:** Results related to postmortem contact and linking objects, cross-tabulated by gestational age

		Gestational age (weeks)						
	Total (*n* = 796)	16–19 (*n* = 128)	20–25 (*n* = 193)	26–33 (*n* = 151)	≥ 34 (*n* = 324)			
	*n* (%)	*n* (%)	*n* (%)	*n* (%)	*n* (%)	χ^2^, p*		Cramér’s V
Postmortem contact								
Mother saw the baby	421 (52.9)	37 (28.9)	66 (34.2)	78 (51.7)	240 (74.1)	115.06,	*p* < 0.001	.380
Father/partner saw the baby^[1]^	469 (58.9)	33 (25.8)	62 (32.1)	104 (68.9)	270 (83.3)	201.34,	*p* < 0.001	.503
Family member/ friend saw the baby^[1]^	276 (34.7)	14 (10.9)	33 (17.1)	45 (29.8)	184 (56.8)	129.70,	*p* < 0.001	.404
Mother touched or held the baby	323 (41.3)	16 (12.5)	46 (24.1)	56 (37.8)	205 (65.1)	141.34,	*p* < 0.001	.425
Mother held the baby	275 (35.1)	10 (7.8)	39 (20.4)	47 (31.5)	179 (56.6)	125.17,	*p* < 0.001	.400
Linking objects								
At least one linking object	242 (30.4)	17 (13.3)	38 (19.7)	42 (27.8)	145 (44.8)	60.21,	*p* < 0.001	.275
Have a photograph	97 (12.2)	7 (5.5)	10 (5.2)	21 (13.9)	59 (18.2)	25.65,	*p* < 0.001	.180
Postmortem contact & linking objects								
Postmortem contact &1 or more linking obj.	198 (24.9)	11 (8.6)	25 (13.0)	33 (21.9)	129 (39.8)	72.77,	*p* < 0.001	.301
No postmortem contact or linking object	331 (41.6)	85 (66.4)	114 (59.1)	64 (42.4)	68 (21.0)	113.37,	*p* < 0.001	.377

*Pearson’s Chi-squared, significance level set at *p* <0.05

^[1]^Data on partners and family members (e.g. seeing/holding) is based on the mother’s testimony

In total, 41.3% of mothers had some physical contact (touched or held) with their baby. Physical contact was significantly associated to gestational age (*p* < 0.001), with a very large effect size: from 16 to 19 weeks only 12.5% of mothers had some physical contact compared to 65.1% of cases ≥ 34 weeks. 35.1% of mothers held their baby, which was also associated to gestational age (*p* < 0.001), with a large effect size (see Table 2).

30.4% of women stated that they left the hospital with at least one object related to the baby (not including medical paperwork) or photograph. There was a significant association between having linking objects and gestational age (*p* < 0.001), showing a medium effect size. Across the whole sample, the percentage of mothers who left the hospital with a photograph was 12.2% (see Table 2).

In total, 24.9% of women had postmortem contact and left the hospital with at least one linking object, while 41.6% had neither postmortem contact nor any linking object; both results were significantly related to gestational age (*p* < 0.001). The highest proportion of women who had both contact and at least one linking object was 39.8% in cases ≥ 34 weeks gestation. The largest proportion of women who had neither postmortem contact nor any linking object was 66.4% for those whose baby died from 16 to 19 weeks gestation.

### Disposition of the body/remains

34.3% of respondents choose the response option ‘no one’ when asked to state who had talked to them about options for the disposition of the body/remains, however it was evident from open-ended responses that some parents also sought out information. Not being spoken to about options for disposition was significantly associated to gestational age (*p* < 0.001), with a very large effect size (see Table 3). Before 26 weeks, almost two-thirds (64.5%) of women reported that ‘no one’ spoke to them about disposition, though in cases from 26 to 33 weeks (13.2%) and ≥ 34 weeks (14.2%) a substantial proportion of respondents also made this claim.

**Table 3:** Results related to the disposition of the body/ remains, cross-tabulated by gestational age

		Gestational age (weeks)						
	Total (*n* = 796)	16–19 (*n =* 128)	20–25 (*n =* 193)	26–33 (*n =* 151)	≥ 34 (*n =* 324)			
	*n* (%)	*n* (%)	*n* (%)	*n* (%)	*n* (%)	χ^2^, p*		Cramér’s V
Communication of procedures and options for the disposition of the body***								
“No one” explained options for disposition	273 (34.3)	96 (75.0)	111 (57.5)	20 (13.2)	46 (14.2)	228.05,	*p* < 0.001	.535
A doctor	240 (30.2)	18 (14.1)	46 (23.8)	59 (39.1)	117 (36.1)	30.56,	*p* < 0.001	.196
A midwife/ nurse	156 (19.6)	11 (8.6)	24 (12.4)	42 (27.8)	79 (24.4)	27.29,	*p* < 0.001	.185
Representative of a funeral home	124 (15.6)	1 (0.8)	6 (3.1)	28 (18.5)	89 (27.5)	**		
Administrator/ social worker/ porter/ other HP	51 (6.4)	2 (1.6)	8 (4.1)	17 (11.3)	24 (7.4)	**		
Don’t know as a family member took charge	9 (1.1)	0 (0.0)	0 (0.0)	3 (2.0)	6 (1.9)	**		
Other	3 (0.4)	0 (0.0)	2 (1.0)	0 (0.0)	1 (0.3)	**		
Not stated	16 (2.0)	2 (1.6)	3 (1.6)	3 (2.0)	8 (2.5)	**		
Disposition method								
Donated to research	92 (11.6)	6 (4.7)	27 (14.0)	27 (17.9)	32 (9.9)	13.83,	*p* = 0.003	.132
Private (burial or cremation)	310 (39.5)	5 (4.0)	19 (10.0)	67 (44.4)	219 (67.6)	248.52,	*p* < 0.001	.559
Hospital managed disposition (incineration)	371 (46.6)	113 (88.3)	144 (74.6)	50 (33.1)	64 (19.8)	255.09,	*p* < 0.001	.566
Burial common plot	12 (1.5)	1 (0.8)	0 (0.0)	6 (4.0)	5 (1.5)	**		
Other (e.g. haemorrhage at home)	11 (1.4)	3 (2.3)	3 (1.6)	1 (0.7)	4 (1.2)	**		
Reason for hospital managed disposition/ incineration^[1]***^	(*n =* 371)	(*n* = 113)	(*n* = 144)	(*n* = 50)	(*n* = 64)			
^a^Only option/ told it was protocol early losses	205 (55.3)	94 (83.2)	90 (62.5)	15 (30.0)	6 (9.4)	106.10,	*p* < 0.001	.535
^b^Lack of information/ no information	42 (11.3)	9 (8.0)	18 (12.5)	6 (12.0)	9 (14.1)	1.97,	*p* = 0.579	–
^c^Told: body not returned in cases of autopsy	44 (11.9)	5 (4.4)	15 (10.4)	12 (24.0)	12 (18.8)	16.21,	*p* = 0.001	.209
Seemed like/ was the best option	38 (10.2)	0 (0.0)	6 (4.2)	11 (22.0)	21 (32.8)	**		
Pressured decision/ in a state of shock	24 (6.5)	1 (0.9)	4 (2.8)	6 (12.0)	13 (20.3)	**		
Other^[2]^	23 (6.2)	0 (0.0)	3 (2.1)	6 (12.0)	14 (21.9)	**		
Not stated	35 (9.4)	5 (4.4)	14 (9.7)	5 (10.0)	11 (17.2)	7.85,	*p* = 0.049	.146
^a + b + c^Decision not taken by the parents	285 (76.8)	107 (94.7)	121 (84.0)	31 (62.0)	26 (40.6)	77.7,	*p* < 0.001	.458
As a % of all cases (*n* = 792*)*	285 (35.8)	107 (83.6)	121 (62.7)	31 (20.5)	26 (8.0)	312.01,	*p* < 0.001	.626

*Pearson’s Chi-squared, significance level set at *p* <0.05

**Chi-squared not calculated due to minimum requirement of 5 cases per cell

***Multiple responses permitted except when response was ‘null’

^[1]^Coded from an open-ended question

^[2]^Includes responses such as: ‘not involved in the decision’, ‘expected ashes to be returned’

Almost half (46.6%) of all dispositions were managed by the hospital, which often means incineration with other hospital waste. Before 26 weeks gestation the analysis shows that in 80.1% of cases the disposition was handled by the hospital, compared to 24.0% in cases ≥ 26 weeks. In 38.9% of cases parents made private arrangements for the disposition (*burial or cremation*), though this was very uncommon < 26 weeks gestation, occurring in only 24 cases. More than 1 in 10 bodies (11.6%) were *donated to research*.

In order to establish why some parents chose a hospital disposition as opposed to a private burial or cremation, the women were asked to explain the decision in an open-ended question. The results reveal that in the majority of cases the decision was out of the hands of the parent(s). Of these cases (*n* = 371), 55.3% were told that is was *protocol for early losses* or that there were *no options for private disposition,* while a further 11.3% responded that they simply received *no information* and 11.9% reported that they were told that *in cases of autopsy the body can’t be returned*. Only 10.2% of those that had a hospital managed disposition stated that they thought it was the best option available. Consequently, some 35.8% of *all* cases were effectively given no choice or denied any possibility of making private arrangements, which is significantly associated with gestational age (*p* < 0.001), showing a very large effect size: 70.1% of cases < 26 weeks, yet also 12.0% of cases ≥ 26 weeks gestation.

### Mode of delivery and accompaniment during the birth

As expected, almost all second trimester (< 26 weeks, in this sample) cases had a vaginal delivery. In cases ≥ 26 weeks gestation 76.6% of births were vaginal and 22.9% were by caesarean. In these cases the analysis found a significant difference (*p* < 0.001) in the rate of vaginal delivery between public (82.9%) and private hospitals (57.6%), with a medium to large effect size. Conversely, caesarean delivery in public hospitals was 16.8% and 41.5% in private hospitals. The research also found that 14.2% of all births were vaginal and instrumentalised (16.9% of all vaginal births), which was significantly related to both gestational age (*p* = 0.004) and class of hospital (*p* = 0.001), though the effect size was small for both (see Table 5).

The analysis found that 70.7% of women were accompanied (by a partner, family member or friend) during the delivery, which included 80.2% in the case of vaginal delivery and 21.2% in the case of caesarean delivery (see Table 4). In terms of class of hospital, private hospitals were significantly associated (*p* = 0.002) with lower rates of accompaniment (61.9%) compared to public hospitals (73.6%), though the effect size was small (Table 5).

**Table 4:** Results related to medical and technical aspects of care, cross-tabulated by gestational age

		Gestational age (weeks)						
	Total (*n =* 796)	16–19 (*n =* 128)	20–25 (*n =* 193)	26–33 (*n =* 151)	≥ 3 4 (*n =* 324)			
	*n* (%)	*n* (%)	*n* (%)	*n* (%)	*n* (%)	χ^2^, p*		Cramér’s V
Mode of delivery								
Vaginal (total)	667 (83.4)	120 (93.8)	183 (94.8)	122 (80.8)	242 (74.7)	47.38,	*p* < 0.001	.244
Vaginal - induced	578 (72.6)	99 (77.3)	152 (78.8)	115 (76.2)	212 (65.4)	14.46,	*p* = 0.002	.135
Vaginal - instrumentalised	113 (14.2)	26 (20.5)	26 (13.5)	9 (6.0)	52 (16.1)	13.53,	*p* = 0.004	.131
Vaginal - natural	76 (9.5)	16 (12.5)	28 (14.5)	7 (4.6)	25 (7.7)	12.26,	*p* = 0.007	.124
Caesarean section (total)	118 (14.8)	0 (0.0)	9 (4.7)	29 (19.2)	80 (24.7)	65.34,	*p* < 0.001	.286
Planned caesarean	22 (2.8)	0 (0.0)	3 (1.6)	4 (2.6)	15 (4.6)	^**^		
Emergency caesarean	80 (10.1)	0 (0.0)	6 (3.1)	22 (14.6)	52 (16.0)	^**^		
Due to failed induction	16 (2.0)	0 (0.0)	0 (0.0)	3 (2.0)	13 (4.0)	^**^		
Other	9 (1.1)	7 (5.5)	1 (0.5)	0 (0.0)	1 (0.3)	^**^		
Not stated	2 (0.3)	1 (0.8)	0 (0.0)	0 (0.0)	1 (0.3)	^**^		
Accompaniment during the birth								
Accompanied during the birth (total)^[1]^	561 (70.7)	84 (66.1)	128 (66.3)	107 (70.9)	242 (74.9)	5.83,	*p* = 0.120	–
Vaginal births	534 (80.2)	82 (68.3)	126 (68.9)	103 (84.4)	223 (92.5)	49.89,	*p* < 0.001	.274
Caesarean births	25 (21.2)	0 (0.0)	2 (22.2)	4 (23.4)	19 (23.8)	^**^		
Partner not allowed to accompany (total)^[2]^	138 (17.3)	26 (20.3)	38 (19.7)	25 (16.6)	49 (15.1)	2.70,	*p* = 0.439	–
Vaginal births	80 (12.0)	24 (20.0)	35 (19.1)	11 (9.0)	10 (4.1)	31.30,	*p* < 0.001	.217
Caesarean births	54 (45.8)	0 (0.0)	3 (33.3)	14 (48.3)	37 (46.2)	^**^		
Explanation/ offer of pathology studies^***^								
“No one” spoke about pathology	171 (21.5)	50 (39.1)	62 (32.1)	21 (13.9)	38 (11.7)	59.82,	*p* < 0.001	.274
Doctor spoke about pathology	524 (65.8)	72 (56.2)	117 (60.6)	107 (70.9)	228 (70.4)	12.22,	*p* = 0.007	.124
Nurse/ midwife spoke about pathology	120 (15.1)	6 (4.7)	16 (8.3)	28 (18.5)	70 (21.6)	29.94,	*p* < 0.001	.194
Other HP spoke about pathology	19 (2.4)	1 (0.8)	6 (3.1)	3 (2.0)	9 (2.8)	^**^		
Not stated	9 (1.1)	0 (0.0)	0 (0.0)	2 (1.3)	7 (2.2)	^**^		
Pathology studies conducted								
At least one pathology study/test conducted	667 (85.5)	89 (71.8)	155 (83.3)	134 (91.2)	289 (89.5)	27.48,	*p* < 0.001	.188
General autopsy	479 (61.4)	50 (40.3)	103 (55.4)	109 (74.1)	217 (67.2)	40.74,	*p* < 0.001	.229
Stillbirths	396 (66.8)	31 (41.3)	57 (66.3)	101 (79.5)	207 (67.9)	31.37,	*p* < 0.001	.230
Termination of pregnancy	104 (55.6)	30 (61.2)	54 (54.0)	12 (60.0)	8 (44.4)	1.80,	*p* = 0.620	–
Placental autopsy	299 (37.6)	27 (21.1)	66 (34.2)	72 (47.7)	134 (41.4)	24.31,	*p* < 0.001	.175
Not stated	16 (2.0)	4 (3.1)	7 (3.6)	4 (2.6)	1 (0.3)	8.44,	*p* = 0.038	.103

*Pearson’s Chi-squared, significance level set at *p* <0.05

^**^Chi-squared not calculated due to minimum requirement of 5 cases per cell

^***^Multiple responses permitted except when response was ‘null’

^[1]^There were two missing cases in the accompaniment variable

^[2]^Other reasons for not being permitted accompaniment during the birth included: Under anaesthesia, surgery, emergency caesarean (22.1%), Respondent wanted to be alone or her partner didn’t want to enter (8.9%) and Other: partner didn’t arrive on time, single mother, etc. (8.5%)

In a closed-end question, respondents were asked to state why they were unaccompanied during the delivery. The analysis found that 17.3% of all respondents stated that their partner/family member was *not allowed* to enter the delivery room, which was not significantly associated to class of hospital or gestational age. Hence, according to respondents’ understandings of what happened, 59.2% of all unaccompanied births (*n* = 139) were accounted for by decisions taken by the HPs, as opposed to the circumstances of the medical treatment, the preferences of the mother/partner or for other reasons (see footnote Table 4).

### Administration of sedatives/ tranquilizers (benzodiazepines)

Respondents were asked if they had been administered sedatives *or* tranquilizers (defined as medication that sedated or tranquilised *not* analgesics, epidural anaesthetics or sleeping aids). The analysis found that 48.0% of women recalled being given sedatives/tranquilizers on at least one occasion, while 7.7% were given such medication twice or more (see Table 5). Administration of sedatives/tranquilizers was not significantly associated with gestational age or type of hospital.

**Table 5:** Results related to medical/technical aspects of care, cross-tabulated by type of hospital

		Type of hospital				
	Total (*n =* 796)	Public (*n* = 593)	Private (*n* = 203)			
	*n* (%)	*n* (%)	*n* (%)	χ^2^, p^*^		Phi
Mode of delivery						
Vaginal (total)	667 (83.8)	521 (87.9)	146 (71.9)	28.29,	*p <* 0.001	−.189
Vaginal - induced	578 (72.6)	449 (86.2)	129 (88.4)	*.46,*	*p =* 0.494	–
Vaginal - instrumentalised	113 (16.9)	73 (14.0)	40 (27.4)	*14.52,*	*p <* 0.001	.148
Vaginal - natural	76 (11.4)	63 (12.1)	13 (8.9)	*1.14,*	*p =* 0.284	–
Caesarean section (total)	118 (14.8)	67 (11.3)	51 (25.1)	22.89,	*p <* 0.001	.170
Planned caesarean	22 (2.8)	10 (1.7)	12 (5.9)	*10.04,*	*p =* 0.002	.112
Emergency caesarean	80 (10.1)	49 (8.3)	31 (15.3)	*8.21,*	*p =* 0.004	.102
Due to failed induction	16 (2.0)	8 (1.3)	8 (3.9)	*5.16,*	*p =* 0.023	.080
Other	9 (1.1)	3 (0.5)	6 (3.0)	^**^		
Not stated	2 (0.3)	2 (0.3)	0 (0.0)	^**^		
Mode of delivery ≥26 weeks gestation	(*n* = 475)	(*n* = 357)	(*n* = 118)			
Vaginal	364 (76.6)	296 (82.9)	68 (57.6)	31.61,	*p <* 0.001	−.358
Caesarean section	109 (22.9)	60 (16.8)	49 (41.5)	30.64,	*p <* 0.001	.254
Accompaniment during the birth						
Accompanied during the birth (total)^[1]^	561 (70.7)	436 (73.6)	125 (61.9)	10.05,	*p =* 0.002	−.113
Vaginal births	534 (80.2)	427 (82.0)	107 (73.8)	*4.76,*	*p =* 0.029	−.085
Caesarean births	25 (21.2)	9 (13.4)	16 (31.4)	*5.58,*	*p =* 0.018	.217
Partner/other not allowed to accompany (total)^[2]^	138 (17.3)	94 (15.9)	44 (21.7)	3.58,	*p =* 0.059	–
Vaginal births	80 (12.0)	57 (10.9)	23 (15.8)	*2.50,*	*p =* 0.114	–
Caesarean births	54 (45.8)	34 (50.7)	20 (39.2)	*1.55,*	*p =* 0.213	–
Administration of sedatives/ benzodiazepines^***^						
Administered at least once	382 (48.0)	274 (46.2)	108 (53.2)	2.96,	*p =* 0.085	–
Administered more than once	61 (7.7)	41 (6.9)	20 (9.9)	1.84,	*p =* 0.174	–
Reason for taking sedatives						
Patient asked for something to help her relax	130 (34.0)	96 (35.0)	34 (31.5)	*.436,*	*p =* 0.509	–
HP told patient to take something to relax	203 (53.1)	143 (52.2)	60 (55.6)	*.352,*	*p =* 0.553	–
HP administered without consulting	68 (17.8)	49 (17.9)	19 (17.6)	*.004,*	*p =* 0.947	–
Effects of sedatives explained at 1st admin. (partially or fully)^[3]^	167 (43.8)	117 (42.7)	50 (46.7)	*.507,*	*p =* 0.476	–
Pathology studies conducted						
At least one pathology study/test conducted	667 (85.5)	519 (89.2)	148 (74.7)	24.82,	*p <* 0.001	.178
General autopsy	479 (61.4)	387 (66.5)	92 (46.5)	25.01,	*p <* 0.001	−.179
Stillbirths	396 (66.8)	314 (71.5)	82 (53.2)	17.17,	*p <* 0.001	−.170
Termination of pregnancy	83 (44.4)	73 (51.0)	10 (22.7)	10.93,	*p* = 0.001	−.242
Placental autopsy	301 (38.6)	230 (39.5)	71 (35.9)	.835,	*p* = 0.361	–
Not stated	16 (2.0)	11 (1.9)	5 (2.5)	.284,	*p =* 0.284	–

^*^Pearson’s Chi-squared, significance level set at *p* <0.05

^**^Chi-squared not calculated due to minimum requirement of 5 cases per cell

^***^Multiple responses permitted - Responding to reasons for administration of sedatives on up to 3 occasions (After diagnosis/ before or at the start of labour, around the time of the birth, after the birth)

^[1]^There were two missing cases in the accompaniment variable

^[2]^Other reasons for not being permitting accompaniment during the birth included: Under anaesthesia, surgery, emergency caesarean (22.1%), Respondent wanted to be alone or her partner didn’t want to enter (8.9%) and Other: partner didn’t arrive on time, single mother, etc. (8.5%)

^[3]^Only respondents who didn’t receive an explanation on all the first occasion they were administered sedatives

HPs appear to be the primary drivers behind the administration of sedatives/tranquilisers: 53.1% of respondents stated that they took sedatives/tranquilisers because the HP *told me it would be better if I took something to help me relax*, while 17.8% responded that HPs *gave me sedatives/tranquilizers without consulting me*. A third (34.0%) selected the option *I asked for something to help me relax*. 43.8% of respondents who took sedatives stated that they couldn’t recall receiving any explanation about their effects from HPs on the first (or only) occasion that they were administered. There were no significant relationships to gestational age or class of hospital.

### Autopsy & pathology services

When asked to state, in a closed-end question, who had explained the options for conducting pathology tests, 21.5% of women choose the response option ‘no one’, though it is apparent from open-ended responses that some respondents subsequently sought out information and/or demanded tests. Whether or not respondents were spoken to about the possibility of conducting pathology studies was significantly associated to gestational age (*p* < 0.001), showing a medium effect size: from 16 to 19 weeks gestation, 39.1% of women stated that ‘no one’ explained options for pathology studies compared to 11.7% in cases ≥ 34 weeks (see Table 4). While *doctors* were the main source of information about pathology studies (65.8% of all cases), respondents also recalled receiving information from *nurses/ midwives* (15.1% of all cases).

At least one pathology study was conducted in 85.5% of cases (Table 5). Losses at earlier gestational ages are associated with having fewer pathology studies (*p* < 0.001), as are cases that were attended in private hospitals (*p* < 0.001). Across the whole sample, a general autopsy was carried out in 61.4% of cases, and was dependent on gestational age (*p* < 0.001), with a medium effect size (Table 4), and class of hospital (*p* < 0.001), with a small effect size (Table 5). The autopsy rate amongst the stillbirths subgroup was 66.8% compared to 44.4% in cases of termination of pregnancy, both showing significant association to gestational age and type of hospital.

The highest autopsy rate was amongst stillbirths in the gestational age range 26–33 weeks (79.5%) and lowest in cases from 16 to 19 weeks (41.3%). In public hospitals, an autopsy was carried out in 71.5% of stillbirth cases and 51.0% of terminations, whereas in private hospitals an autopsy was conducted in 53.2% of stillbirths and 22.7% of terminations (Table 5). The stillbirth autopsy rate (cases ≥ 20 weeks, *n* = 518, not-stated = 9, excluding terminations) was 70.5% for the whole sample, or 75.4% in public hospitals and 55.5% in private hospitals. Finally, respondents recalled that a placental autopsy was conducted in 37.6% of cases, which was significantly associated with gestational age (*p* < 0.001), with a medium effect size, but not to class of hospital.

## Discussion

With a non-representative technique where respondents self-select some important limitations, such as coverage and representativeness of the sample, restrict the generalizability of the results [28]. Analogous to other studies in this area it is apparent that the sample is skewed toward the middle and higher educated social classes [29, 30], as well as Spanish nationals. However, as detailed below, both the autopsy and caesarean rate correspond to national data, increasing confidence in the findings. In terms of memory as a source of error, the sample was restricted to deaths occurring 60 months prior to completion of the survey, which appears to be an acceptable duration from event to recall in obstetrics and labour [31, 32]. However, the effect of trauma on memory may be a negative factor, as might the administration of benzodiazepines, which are known to impair memory [33].

### Bereavement support care

In settings where parents are supported to see and hold their baby after intrauterine death very few choose not to do so [29, 34, 35]. Bereavement support care is a relatively new phenomenon in Spanish hospitals and, by comparison to other high-income countries, levels of postmortem contact are low, being no higher than 74.1% ≥34 weeks gestation, and less than one-in-three in mid-to-late second trimester deaths (16–25 weeks). Levels of physical contact with the baby are also very low when contrasted to other countries. Postmortem contact is considered a key component of care by virtue of aiding the important process of reconstruction and integration into the family and social network, as well as facilitating the creation of positive memories about the birth and giving parents an opportunity to continue parenting their child [8, 35–37]. Most importantly, parents are overwhelmingly positive about such experiences [8, 29, 36], though this is not to say that parents don’t describe negative experiences, a small number do [36].

Closely related to postmortem contact, linking objects and photographs are highly valued by parents [1]. They play a crucial role in the process of grief by assisting memory making, meaning reconstruction and continued bonds [38, 39]. This study found that only a small proportion of parents left the hospital with a linking object (30.4%). Research in Spain suggests that training for HPs may be a significant barrier to the implementation of these practices [40], but the results indicate that a significant opportunity exists for an improvement to care quality with resultant benefits for parents.

Options for sensitive and respectful disposal of the body or remains should be available for all parents, regardless of type of death or gestational age [41, 15]. When parents are not fully involved in such decisions, or denied autonomy, it can be extremely distressful and associated with feelings of guilt, particularly if parents aren’t sure how and where their child’s body was disposed of, or have regrets about the decision [1, 9, 15, 42]. 70.1% of cases <26 weeks gestation and 12.0% of cases ≥26 weeks gestation reported that HPs told them (or in a few cases simply provided no information) that a hospital managed disposal was the only option available, effectively denying any possibility of autonomous decision-making and a private arrangement. This should not be interpreted as meaning that all these women and their partners would have chosen a private arrangement, but the result supports qualitative research findings [9] and anecdotal evidence that many women are explicitly denied this possibility. The more surprising result is that such a substantial number of women reported similar experiences in cases ≥ 26 weeks gestation.

In Spanish hospitals, such protocols appear to originate in interpretations of a national law related to the legal responsibility to register all fetal deaths ≥ 26 weeks gestation (Law of the 15th of June 1957) as meaning that the hospital is obliged to handle the disposition, regardless of parents’ wishes. In February 2016 the Spanish Constitutional Tribunal ruled this interpretation to be erroneous and that no hospital protocol supersedes the individual rights of parents to a respectful disposition for their baby, regardless of gestational age [43]. Addressing these issues and providing options for respectful disposition poses a challenge for hospital administrators and HPs, but should be seen as a way to improve parents’ experiences, reduce distress and to make a positive contribution to the grief process.

### Medical and technical aspects of care

Vaginal delivery is recommended in all but the most unusual cases of stillbirth, where clinical indications exist or because of the preference of the patient [12, 18]. Caesarean section is associated with short and long-term maternal morbidity, including an increased possibility of uterine rupture in future induced births [44–46], and fetal mortality in future pregnancies [47]. For births ≥ 26 weeks gestation, the study found an overall caesarean rate of 22.9%. Focusing only on stillbirths ≥ 20 weeks the caesarean rate was 20.7%, which corresponds to national data for the same period as this study [48]. Although we shouldn’t jump to any immediate conclusions about this finding, as the clinical circumstances of each case are unknown, the caesarean rate seems particular high, almost double that found in other studies [49–51]. However, the new finding here is the large variation between private and public hospitals; in cases ≥ 26 weeks, 41.5% of women treated in private hospitals gave birth by caesarean, compared to 16.8% in public hospitals, a result that coincides with differences in caesarean rates in live births in Spanish hospitals [52].

Although many of the women reported that the caesarean was performed due to emergency, only 21 deaths were classified as occurring during the birth, suggesting that the definition of ‘emergency’ may often not be in the clinical sense. Research has found that many factors may drive women’s demand for an elective caesarean after stillbirth. They may feel fearful for their own safety and their partners may share this fear [10, 53, 54], they may also harbour hopes that a quick birth and resuscitation might save the baby [55]. It may also be one of the few ways that women feel they can exercise some control over their situation [55]. However, when supported and given good information about potential physiological, psychological and social benefits most women see the value in a vaginal birth [56], and many have positive and valuable experiences [57], though it is manifestly important that women feel in control of the decision-making process and that the mode of delivery isn’t imposed.

Accompaniment by a partner, family member or friend have added importance following intrauterine death, not only for the support that they may provide during a stressful experience, but also for participation in decision-making and shared experiences of ritual such as postmortem contact. In this study, almost a third of mothers in the sample were alone during such a difficult and traumatic experience, substantially higher than the 10% reported in a U.K. study with a similar methodology [49]. More than half of this subgroup of respondents (17.3% of the total sample) reported that the main reason they were alone was because the HPs wouldn’t permit their partner to enter the delivery room. Increasing rates of accompaniment should have emotional benefits for women, and may also help reduce medical interventions, as has been found in live births [58].

The administration of benzodiazepines as a treatment for grief, depression or trauma in the few days after bereavement has not been widely studied, but there is no solid evidence for any benefits to the patient in terms of sleep or grief [22]. The finding that almost half of the women in the sample were prescribed sedatives/tranquilizers during the hospital stay is in line with the findings of a 1994 U.K. study [25], but is 4 to 5 times higher than that reported in a more recent study in the U.S. [23]. As the clinical circumstances are unknown, it is difficult to reach any evaluation, however, it should be borne in mind that although a substantial proportion of women (34.0%) asked for something to help them relax, the balance reported taking sedatives on the advice of the attending HPs. Additionally, more than half of those administered sedatives couldn’t recall receiving an explanation about the effects of the medication prior to taking them. The authors of the U.S. study highlight that there is little evidence of long-term dependence on benzodiazepines following administration after perinatal death, however, without any strong supporting evidence their use shouldn’t be routine. Furthermore, their potential impact on memory [33] may be important as memories of the hospital stay and postmortem contact have an important function in the process of grief.

Perinatal autopsy and other pathology studies can have important emotional benefits for parents and the grief process and help reduce feelings of guilt [57, 59], as well as providing new and valuable information about diagnosis and cause of death, which may alter clinical management in a subsequent pregnancy [60]. When conducted with an appropriate protocol and classification system, the perinatal autopsy, including placental examination, cytogenetic, biochemical and other tests, can find a cause of death in up to 75% of cases [60, 61].

In this study, no pathology studies were conducted in 14.5% of cases, being as high as 1 in 3 cases < 20 weeks gestation. The stillbirth autopsy rate (≥ 20 weeks) of 70.5% corresponds to national data for the same period [48] and is at the higher end of rates reported in other countries [21]. However, significant differences exist between public (75.4%) and private hospitals (55.5%) and with intrauterine deaths < 20 weeks gestation (40.3%) and terminations of pregnancy (44.4%), where autopsy may also provide valuable additional information [62, 63]. Although, only 37.6% of respondents reported that a placental autopsy had been conducted, it may be that respondent’s knowledge of the details of the autopsy report is not accurate and suggests that information and communication about autopsy practices and findings could be improved.

## Conclusions

In general the study reveals that practices in perinatal bereavement care in Spanish hospitals show substantial differences to other countries with similar economies, a finding that also emerged from a recent international study [64]. This is not particularly surprising as this is an emerging field in Spain, while other high-income countries have been developing perinatal bereavement care since the 1970s. Regardless, there are also well-established standards for bereavement support care and physiological management of women who have experienced an intrauterine death [1, 12, 16]. Such care is widely recognised as having psychosocial benefits for women and their families, and in this respect the results highlight that strategies to improve care should be a high priority, as poor care can have negative long-term effects on health and grief. All women and their partners should have opportunities for postmortem contact and to keep linking objects, to conduct an autopsy, to arrange a respectful disposition, to receive evidence-based information when deciding on the mode of delivery and no woman should be obliged to give birth alone. Overall, a shift to a more psychosocial focused care could have very positive impacts on perceptions of care quality, as well as making an important positive contribution to experiences of grief at such a difficult time in the lives of these women and their families.

## Additional files

Additional file 1: Questionnaire (Spanish). (PDF 425 kb)

Additional file 2: Questionnaire (English). (PDF 394 kb)

Additional file 3: Additional details on the study methodology. (PDF 172 kb)

## Abbreviations

HP: Health professional
