# Supplementary material for: Care quality following intrauterine death in Spanish hospitals: results from an online survey
Source: BMC Pregnancy Childbirth. 2018 Jan 10;18:22. doi: 10.1186/s12884-017-1630-z (PMC5763533; doi:10.1186/s12884-017-1630-z)
Supplement: Supplementary file 1 — Questionnaire (Spanish). (PDF 425 kb) [file 12884_2017_1630_MOESM1_ESM.pdf]

ADDITIONAL FILE 2.
QUESTIONNAIRE (SPANISH)

ARTICLE TITLE
Care quality following intrauterine death in Spanish hospitals: Results from an online survey

AUTHOR
Paul Richard Cassidy BBS MPhil

Doctoral student
Universidad Complutense de Madrid
Facultad de Ciencia Política y Sociología
Somosaguas, Pozuelo de Alarcon
Madrid, 28223
Email: pcassidy@ucm.es

Researcher
Umamanita (Stillbirth Charity)
C/ Hierbabuena 15, Esc B, 4 Izq
Madrid, 28039
Email: paulcassidy@umamanita.es

ENCUESTA A MADRES SOBRE SUS EXPERIENCIAS EN EL SISTEMA SANITARIO ESPAÑOL DESPUÉS DE LA MUERTE PERINATAL

SOBRE LA ENCUESTA:

- El objetivo de este estudio es mejorar nuestra comprensión de las experiencias de madres y padres en el hospital durante y después de una muerte gestacional ocurrida a partir de la semana 16.

- El cuestionario ha sido desarrollado por Umamanita, una asociación sin ánimo de lucro, que apoya a las madres y padres después de una muerte perinatal.

¿PARA QUIÉN ES EL CUESTIONARIO?

- Aunque entendemos que no importan las semanas de gestación por razones relacionadas con los procesos hospitalarios, este cuestionario está destinado solamente a madres que han sufrido una pérdida desde la semana 16 del embarazo en adelante e incluye las ocurridas durante el parto. Si tu bebé nació vivo/a, este cuestionario no es aplicable. Contempla los casos de muertes espontáneas, terminación terapéutica del embarazo por problemas del feto o amenaza para la salud materna y reducción selectiva en embarazos múltiples.

- Aunque el cuestionario está dirigido sólo a las madres, no se excluye la posibilidad de que tu pareja pueda ayudarte a completarlo ya que entendemos que muchas veces, en el hospital, la pareja por encargarse de ciertos trámites, ha podido estar expuesta a otras experiencias o puede tener recuerdos más nítidos de ciertos momentos.

- Si has tenido más de una pérdida, por favor contesta en base a tus experiencias de la última pérdida.

- Para pérdidas múltiples, ocurridas en el mismo embarazo, tan sólo se debe contestar un cuestionario, no varios.

INFORMACIÓN IMPORTANTE ANTES DE RELLENAR EL CUESTIONARIO:

- Somos conscientes de que la encuesta trata asuntos emocionalmente difíciles y por ello te agradecemos mucho tu participación. Nuestro compromiso es utilizar los resultados de la encuesta como base para promover mejoras en los cuidados.

- Es importante recordar que no hay respuestas correctas ni erróneas, sólo existe tu opinión personal, por ello es muy importante para la fiabilidad de la encuesta que te centres sólo en tus propias experiencias.
- Si hay una pregunta que no entiendes o crees que no puedes contestarla bien, no te preocupes, déjala en blanco y pasa a la siguiente.

- El cuestionario debería llevar unos 45 minutos, lo óptimo es rellenarlo en una sola vez pero si tienes que parar y volver no hay problema, simplemente deja el cuestionario abierto en el explorador/ordenador.

- El cuestionario es anónimo, recuerda que todas tus respuestas y datos serán tratados con fines estadísticos y nunca de forma individual, así que te garantizamos absoluta confidencialidad de acuerdo con la Ley de Protección de Datos de Carácter Personal 15/99.

- Si tienes alguna duda acerca del cuestionario y te gustaría aclararla puedes contactar con Paul Cassidy por e-mail: paulcassidy@umamanita.es.

Antes de empezar queremos hacerte una serie de preguntas que nos ayudarán a clasificar tus respuestas.

1 ¿En qué provincia vives ahora?

2 ¿En qué provincia vivías cuando tuviste la pérdida?

3A ¿El hospital donde estabas ingresada era privado o público?

Hospital público .... 1 → P3C
Hospital privado .... 2 → P3C
Primero privado y luego público 3

Si fue trasladada de un hospital privado a uno público

3B ¿En qué momento te trasladaron al hospital público?

Después del diagnóstico 1
Después del parto 2
Otro, especificar por favor 3

3C ¿Cuál es el nombre del hospital donde estuviste ingresada? Si estuviste en más de un hospital elige el hospital donde pasaste más tiempo (noches ingresadas).

4 ¿Cuántos años tienes?

5 ¿Cuál es tu nacionalidad?

6 ¿Cuál de las siguientes opciones describe mejor tu nivel de estudios?

No sé leer o escribir (cuestionario cumplido con asistencia) 1
Fui menos de 5 años a la escuela 2
Fui a la escuela 5 años o más pero sin completar EGB, ESO o Bachillerato elemental 3
Bachiller elemental, EGB o ESO completa (Graduado escolar) 4
Bachiller superior, BUP, Bachiller LOGSE, COU, PREU 5
FPI, FP grado medio, Oficialía industrial o equivalente 6
FPII, FP superior, Maestría industrial o equivalente 7
Diplomatura, Arquitectura o Ingeniería técnica; 3 cursos aprobados de Arquitectura, Ingeniería o equivalente 8
Licenciatura o equivalente 9
Máster o Doctorado 10

7 ¿Cuál de las siguientes opciones describe mejor tu ocupación?

Profesional, técnico 1
Directivo de la administración pública y de empresas .. 2
Personal administrativo 3
Comerciante y vendedor 4
Personal de servicios 5
Agricultor, ganadero, arboricultor, pescador y cazador 6
Trabajador de la producción, conductores de equipos de transportes y peones (no agrarios) 7
Profesional de las fuerzas armadas 8
Estudiante 9
Persona dedicada a las labores de su hogar 10
Jubilado, retirado, pensionista y rentista 11
Persona que no puede ser clasificada 12

8 ¿Cuál es tu estado civil actual?

Casada / pareja de hecho / cohabitando con pareja ... 1
Soltera 2
Viuda 3
Separada 4
Divorciada 5
Divorciada y nuevamente casada / pareja de hecho / cohabitando con pareja 6

9 ¿Cuál era tu estado civil cuando tuviste la pérdida?

Casada / pareja de hecho / cohabitando con pareja ... 1
Soltera 2
Viuda 3
Separada 4
Divorciada 5

10 ¿Qué tipo de embarazo tuviste?

Singular 1
Gemelar / mellizos 2
Trillizos 3
Cuatrillizos o más 4

11 ¿Cuál de las siguientes opciones describe mejor el tipo de pérdida que tuviste?

Pérdida espontánea/muerte súbita intrauterina 1
Terminación terapéutica del embarazo por problemas del bebé 2
Terminación terapéutica del embarazo por amenaza para la salud materna 3
Reducción selectiva en embarazos múltiples 4
Neonatal 5
Muerte durante el parto 6
Otro, especificar por favor 7

12 ¿Por favor, nos podrías indicar en qué momento del embarazo murió tu bebé/s?

Entre la semana 16 y 19 1
Entre la semana 20 y 21 2
Entre la semana 22 y 25 3
Entre la semana 26 y la 29 4
Entre la semana 30 y la 33 5
Entre la semana 34 y la 36 6
Entre la semana 37 y la 41 7
Entre la semana 42 y el parto (no durante el parto) 8
Durante el parto 9

13 ¿Cuántos bebés perdiste?

Chicos *Chicas*

14A ¿En qué mes y año perdiste a tu bebé/s?

Mes *Año*

14B ¿Cuánto hace que sufriste la pérdida?

Durante las últimas 6 semanas 1
Durante los últimos 3 meses (90 días) 2
Entre 4 meses y 6 meses 3
Entre 7 meses y 12 meses 4
Hace más de 12 meses 5

15 ¿Hubo tratamiento de fertilización en la concepción?

Sí 1
No 2

16 ¿Habías tenido previamente alguna de las siguientes pérdidas?
Marca todas las opciones que sean necesarias

No 1
Aborto/s espontáneo/s (hasta la semana 12) 2
Aborto/s espontáneo/s (semana 13 hasta 19) 3
Muerte/s intrauterina/s espontánea/s (semana 20 hasta el parto) 4
Reducción selectiva en embarazos múltiples 5
Interrupción voluntaria del embarazo por problemas del bebé 6
Interrupción voluntaria del embarazo por amenaza de la salud materna 7
Muerte/s neonatal/es (desde nacimiento hasta 28 días) 8
Muerte de un hijo mayor de 28 días 9
Otro, especificar por favor 10

Si la pérdida fue hace más de 3 meses...

17 Estás contestando el cuestionario sobre tu última pérdida entre la semana 16 y el parto. Desde esta última pérdida hasta ahora, ¿has tenido una pérdida más temprana?

No 0
Sí, he tenido una pérdida temprana 1
Sí, he tenido más de una pérdida temprana 2

18 ¿Tenías hijo/as nacidos/as con vida antes de esta pérdida?

Sí 1
No 2

Si la pérdida fue hace más de 6 semanas...

19 ¿Estás embarazada en este momento o has tenido hijos nacidos con vida después de esta última pérdida?
Marca todas las opciones que sean necesarias

Estoy embarazada en este momento 1
Sí, he tenido hijos nacidos con vida 2
No 3

20 ¿Conocías el sexo del bebé/s antes de que te comunicasen que el bebé/s había muerto o antes de su nacimiento?

Sí 1
No .... 2 → P22

21 Si conocías el sexo antes de que te comunicaran que el bebé/s había muerto, ¿ya le habías puesto nombre?

Sí 1
No 2

22 ¿Hubo alguna de las siguientes complicaciones durante el embarazo?
Marca todas las opciones que sean necesarias

No tuve ninguna complicación durante el embarazo .. 1
Diabetes gestacional: exceso de los niveles de azúcar en la sangre durante el embarazo 2
Diabetes mellitus tipo I: Diabetes mellitus insulino-dependiente 3
Hiperémesis gravídica (Hyperemesis gravidarum, HG): Náuseas y vómitos intensos y persistentes durante el embarazo, más extremos que las "náuseas matutinas" 4
Hipertensión (relacionada con el embarazo): Hipertensión que comienza después de 20 semanas de embarazo y desaparece después del parto 5
Anemia: Nivel de glóbulos rojos sanos más bajo de lo normal 6
Desprendimiento placentario: La placenta se separa de la pared uterina antes del parto, lo que puede significar que el bebé no reciba suficiente oxígeno 7
Placenta previa: La placenta cubre toda la apertura del cuello uterino dentro del útero o parte de ella 8
Retraso del crecimiento intrauterino 9
Poco líquido amniótico (oligohidramnios) 10
Corioamnionitis (infección de las membranas placentarias y del líquido amniótico) 11
Problemas de tiroides de la madre 12
Obesidad o sobrepeso importante de la madre 13
Amenaza de parto prematuro 14
Rotura prematura de membranas 15
Cuello uterino incompetente 16
Amenaza de aborto (hasta 20 semanas) 17
Sangrado vaginal 18
Colestasis gravídica 19
Exceso de liquido amniótico 20
Otro, especificar por favor 21

23 ¿Dónde estabas cuando te comunicaron la muerte de tu bebé/s o qué el pronóstico no era bueno?

En una sala de urgencias/UCI 1
En la consulta / despacho del médico 2
En una habitación para familias 3
Reanimación 4
Neonatología/ UCIN 5
UCI 6
Paritorio 7
Ecografía-monitores 8
Centro Salud 9
Habitación 10
Quirófano 11
Sala de dilatación 12
Por teléfono 13
Casa 14
Otro, especificar por favor 15

24 ¿Quién te comunicó las malas noticias?

Un médico 1
Una matrona o enfermera 2
Mi pareja u otro familiar .... 3 → P26
Otro, especificar por favor 4

Si un médico o enfermera comunicó las malas noticias

25 ¿Estabas acompañada cuando te comunicaron la muerte de tu bebé/s o que el pronóstico no era bueno?

Sí, por mi pareja 1
Sí, por un miembro de la familia o amigo . 2
No, estaba sola .... 3 → P27

Si, estaba acompañada

26 ¿Te ofrecieron un sitio privado donde estar con tu pareja o familia para asimilar la noticia?

Sí........ 1
No........ 2

27 Ahora nos gustaría que pensaras en la interacción con los profesionales en el momento que te comunicaron que el bebé/s estaba muerto o que el diagnóstico no era bueno.

Marca el número que mejor indique el grado de acuerdo o desacuerdo actualmente con cada una de ellas. Si no estás segura, usa la categoría "Ni estoy de acuerdo/ni en desacuerdo". Por favor, trata de usar esta categoría cuando verdaderamente no tengas una opinión clara.

1 = Estoy totalmente de acuerdo
2 = Estoy de acuerdo
3 = Ni estoy de acuerdo/ni en desacuerdo
4 = No estoy de acuerdo
5 = Estoy totalmente en desacuerdo

Cuando me presenté en el hospital / centro salud me trataron con prioridad	1	2	3	4	5
Intuí por la reacción del profesional sanitario que el pronóstico no era bueno	1	2	3	4	5
Observando el monitor intuí que el pronóstico no era bueno	1	2	3	4	5
El/la medico tardó mucho tiempo en verme	1	2	3	4	5
Recibí una explicación clara del diagnóstico, en un leguaje fácil de entender	1	2	3	4	5
Tuve la oportunidad de hacer más preguntas sobre el diagnóstico	1	2	3	4	5
La persona que me comunicó las malas noticias era una persona empática y comprensiva	1	2	3	4	5
Me sentí acompañada por los profesionales en el tiempo después de recibir las malas noticias	1	2	3	4	5

28 ¿Cuál de las siguientes opciones describe mejor tu alojamiento?

Habitación privada / no compartida 1
Habitación compartida con otra madre embarazada o en posparto........ 2 → P30
Habitación compartida con una paciente que no era de maternidad........ 3
Sala común........ 4
Urgencias........ 5
Sala dilatación-paritorio........ 6
Primero compartida-después individual 7
Habitación compartida, con otra madre con pérdida........ 8
No hubo ingreso........ 9
Otro, especificar por favor........ 10

29 ¿Desde la habitación donde estabas alojada podías escuchar los llantos de los bebés en la unidad de maternidad?
Marca todas las opciones que sean necesarias

No........ 1
Algo........ 2
Sí........ 3

30 ¿Qué tipo de parto tuviste?

Parto eutócico o espontáneo (parto vaginal que se inicia de forma espontánea, es decir, sin medicación y termina también de forma espontánea, es decir, no es necesario realizar maniobras quirúrgicas para facilitar la expulsión del bebé)........ 1
Parto distócico (parto vaginal en el que se necesitan maniobras o intervenciones quirúrgicas para la finalización del parto, es decir, instrumental para facilitar la expulsión del bebé, lo normal es ventosa, espátulas o fórceps)........ 2
Parto inducido-estimulado (parto vaginal en el que es necesario inducir las contracciones del trabajo de parto mediante medicación: prostaglandinas y/u oxitocina)........ 3
Parto inducido-estimulado y distócico (parto vaginal en el que se inducen las contracciones del trabajo de parto mediante prostaglandinas y/u oxitocina y finaliza el parto necesitando realizar maniobras quirúrgicas para facilitar la expulsión del bebé)......... 4
Cesárea programada........ 5
Cesárea de urgencia........ 6
Cesárea por fallo de inducción........ 7
Otro, especificar por favor........ 8

31 ¿Estuvo alguien contigo durante el parto?

Sí, mi pareja........ 1 → P33
Sí, un familiar o amigo........ 2 → P33
No, estaba sola........ 3

32 Nos has indicado que no estuviste acompañada durante el parto, ¿Por qué?

Lo elegí así........ 1
No había nadie para estar conmigo........ 2
Mi pareja no quiso entrar........ 3
A mi pareja no le dejaron entrar........ 4
Otro, especificar por favor........ 5

33 ¿Hubo alguna complicación durante el parto, aparte de la pérdida?

Sí........ 1
No........ 2

34 ¿Cuántas noches estuviste ingresada en el hospital, contando desde el ingreso hasta el alta?

Menos de 1 día / no pasé la noche........ 1
1-2 noches........ 2
3-4 noches........ 3
5-7 noches........ 4
8 noches-2 semanas........ 5
Más de 2 semanas........ 6

35 ¿Te dieron sedantes o tranquilizantes en algún momento? *(fármacos para sedarte o tranquilizarte NO fármacos para el dolor, analgésico, anestésicos como la epidural o pastillas para dormir)*
Marca todas las opciones que sean necesarias

No........ 1 → P43
Después de la comunicación de las malas noticias o durante la parte inicial del trabajo de parto........ 2
Durante el parto (justo antes o durante el expulsivo)........ 3
Después del parto........ 4

Si te administraron sedantes antes del parto

36 Nos has indicado que te dieron sedantes, después de la comunicación de las malas noticias o durante la parte inicial del trabajo de parto, ¿cuál de las siguientes opciones describe mejor la razón por la que te dieron sedantes en aquel momento?

Pedí que me dieran algo para relajarme.................... 1
Me dijeron que sería mejor que me tomara algo para relajarme.................... 2
Me dieron sedantes sin consultar conmigo................ 3

37 ¿Te explicaron los efectos de los sedantes antes de administrártelos?

Sí, me lo explicaron muy bien.................... 1
Sí, pero no del todo.................... 2
No.................... 3

Si te administraron sedantes durante el parto

38 Nos has indicado que te dieron sedantes, durante el parto (justo antes o durante el expulsivo), ¿cuál de las siguientes opciones describe mejor la razón por la que te dieron sedantes en aquel momento?

Pedí que me dieran algo para relajarme.................... 1
Me dijeron que sería mejor que me tomara algo para relajarme.................... 2
Me dieron sedantes sin consultar conmigo................ 3

39 ¿Te explicaron los efectos de los sedantes antes de administrártelos?

Sí, me lo explicaron muy bien.................... 1
Sí, pero no del todo.................... 2
No.................... 3

Si te administraron sedantes después del parto

40 Nos has indicado que te dieron sedantes, después del parto, ¿cuál de las siguientes opciones describe mejor la razón por la que te dieron sedantes en aquel momento?

Pedí que me dieran algo para relajarme.................... 1
Me dijeron que sería mejor que me tomara algo para relajarme.................... 2
Me dieron sedantes sin consultar conmigo................ 3

41 ¿Te explicaron los efectos de los sedantes antes de administrártelos?

Sí, me lo explicaron muy bien.................... 1
Sí, pero no del todo.................... 2
No.................... 3

42 ¿Sientes que los sedantes afectaron tus recuerdos del tiempo en el hospital?

Sí, mucho (creo que no recuerdo cosas importantes) 1
Sí, un poco (pero creo que recuerdo la mayoría de las cosas importantes).................... 2
No creo que los sedantes me afectaron los recuerdos.................... 3

43 ¿Después del parto, tú o tu pareja (si corresponde) viste/vio al bebé/s?

No.................... 1
Sí, pero yo no, sólo mi pareja.......... 2
Sí, sólo yo.................... 3 → P44 t P50
Sí, yo y mi pareja.................... 4 → P44 t P50

44 ¿Otro familiar o amigo de la familia vio el bebé/s?
Marca todas las opciones que sean necesarias

No, nadie.................... 1
Sí, un abuelo/a u otro familiar.................... 2
Sí, un/a amigo/a.................... 3

Si no viste a tu bebé…

45 Nos has indicado que no viste a tu bebé. Por favor, marca el número que mejor indique el grado de acuerdo o desacuerdo actualmente con cada una de las siguientes frases. Si no estás segura, usa la categoría "Ni estoy de acuerdo/ni en desacuerdo". Por favor, trata de usar esta categoría cuando verdaderamente no tengas una opinión clara.

1 = Estoy totalmente de acuerdo
2 = Estoy de acuerdo
3 = Ni estoy de acuerdo/ni en desacuerdo
4 = No estoy de acuerdo
5 = Estoy totalmente en desacuerdo

Recibí bastante información acerca de la decisión de ver o no ver al bebé/s	1	2	3	4	5
Me aconsejaron que sería mejor que no viera al bebé/s	1	2	3	4	5
Aunque decidí no ver a mi bebé me sentí presionada a verle	1	2	3	4	5
Mi pareja y yo tuvimos opiniones diferentes acerca de ver al bebé/s	1	2	3	4	5
Me arrepiento de no haber visto a mi bebé/s	1	2	3	4	5

46 ¿En algún momento algún profesional te dijo que no podrías ver a tu bebé/s?

No.................... 1
Sí.................... 2 → P49

Si le dijeron que no podría verle

47 Por favor, indícanos quién te/os decía que no podrías ver a tu bebé/s.
Marca todas las opciones que sean necesarias

Un/a médico.................... 1
Un/a matrona/ o enfermera/o.................... 2
Otro, especificar por favor.................... 3

48 ¿Qué razón te dieron para decirte que no podrías ver a tu bebé/s?
Puedes contárnoslo en el espacio de abajo

Si no viste a tu bebé…

49 ¿Hay algo más, que nosotros no hemos preguntado, relacionado con el hecho de *no ver* a tu bebé/s que te gustaría contarnos?
Puedes contárnoslo en el espacio de abajo

Si viste a tu bebé…

50 Nos has indicado que tuviste ocasión de ver a tu bebé/s, ¿dónde le viste?
Marca todas las opciones que sean necesarias

En la habitación donde nos alojaron.................... 1
En una sala de urgencias/UCI.................... 2
En la sala de consultas / despacho del medico.......... 3
En la sala de partos.................... 4
En una sala contigua a la sala de partos.................... 5
En una habitación para familias.................... 6
Neonatología.................... 7
Quirófano/sala contigua.................... 8
Reanimación.................... 9
Otro, especificar, por favor.................... 10

Si viste a tu bebé…

51 ¿Indica si tú o tu pareja hicisteis algo de lo siguiente cuando visteis a tu bebé/s?

Marca todas las opciones que sean necesarias

	Yo	Yo y mi pareja	Mi pareja	No
Tocarle/s	1	2	3	4
Sostenerle/s	1	2	3	4
Vestirle/s o arroparle/s	1	2	3	4
Lavarle/s	1	2	3	4
Velarle/s durante un tiempo	1	2	3	4
Tomar fotografías	1	2	3	4

Si viste a tu bebé…

52 ¿En total, durante cuánto tiempo aproximadamente, estuviste con tu bebé?

1-2 minutos 1
3-5 minutos 2
6-20 minutos 3
21-60 minutos 4
1 hora a 2 horas 5
Más de 2 horas 6
No sé, no puedo decirlo 7

Si viste a tu bebé…

53 Nos has indicado que viste a tu bebé. Por favor, marca el número que mejor indique el grado de acuerdo o desacuerdo actualmente con cada una de las siguientes frases. Si no estás segura, usa la categoría “Ni estoy de acuerdo/ni en desacuerdo”. Por favor, trata de usar esta categoría cuando verdaderamente no tengas una opinión clara.

1 = Estoy totalmente de acuerdo
2 = Estoy de acuerdo
3 = Ni estoy de acuerdo/ni en desacuerdo
4 = No estoy de acuerdo
5 = Estoy totalmente en desacuerdo

Recibí bastante información acerca de la decisión de ver o no ver al bebé/s	1	2	3	4	5
Me aconsejaron que sería mejor que no viera al bebé/s	1	2	3	4	5
Me sentí presionada a ver a mi bebé/s	1	2	3	4	5
Me presentaron al bebé/s de una manera respetuosa y afectuosa	1	2	3	4	5
El sitio donde vi a mi bebé/s fue un lugar privado	1	2	3	4	5
Sentí que podría pasar todo el tiempo que quería con mi bebé	1	2	3	4	5
Los profesionales fueron respetuosos en el trato físico de mi bebé/s	1	2	3	4	5
Los profesionales participaron en el proceso de ver / sostener/ velar al bebé/s	1	2	3	4	5
Mi pareja y yo tuvimos opiniones diferentes acerca de ver al bebé/s	1	2	3	4	5
Fue una buena decisión ver a mi bebé/s	1	2	3	4	5

Si viste a tu bebé...

54 ¿Hay algo más, que nosotros no hemos preguntado, relacionado con el hecho de *ver* a tu bebé/s, que te gustaría contarnos?

Puedes contárnoslo en el espacio de abajo

55 Cuando las enfermeras y/o matronas te hablaron del bebé, ¿cómo se referían a él/ella?

Marca todas las opciones que sean necesarias

Por su nombre 1
El / la bebé 2
El feto 3
Él / ella o se 4
No sé / no recuerdo 5

56 Cuando los/las médicos/as te hablaron del bebé, ¿cómo se referían a él/ella?

Marca todas las opciones que sean necesarias

Por su nombre 1
El / la bebé 2
El feto 3
Él / ella o se 4
No sé / no recuerdo 5

57 ¿Cuáles de los siguientes recuerdos guardaste del hospital?

Marca todas las opciones que sean necesarias

Ninguno 1
Fotografía(s) 2
Impresión de las huellas de las manos / pies 3
Mechón de pelo 4
Pulsera de identificación 5
Ecografía 6
Manta / ropa 7
Pinza del cordón umbilical 8
Informes médicos/historial/autopsia 9
Otro, especificar por favor 10

58 ¿Tienes algún recuerdo físico u objeto del hospital o del embarazo que sea especialmente importante para ti y tu relación con tu bebé?

Puedes contárnoslo en el espacio de abajo

59 ¿Te preguntaron por creencias religiosas o espirituales importantes?

Sí 1
No 2
No sé 3

60 ¿Te ofrecieron la posibilidad de hablar con un/una psicólogo/a formado/a en duelo gestacional / neonatal, durante la estancia hospitalaria?

Sí 1
No .... 2 → P62

Si le ofrecieron la posibilidad de apoyo psicológico

61 ¿Aceptaste la oferta de hablar con el/la psicólogo/a o psiquiatra?

Sí 1
No 2

62 Ahora nos gustaría que pensaras en las relaciones con los médicos, matronas y enfermeras. Por favor, marca el número que mejor indique el grado de acuerdo o desacuerdo actualmente con cada una de las siguientes frases. Si no estás segura, usa la categoría “Ni estoy de acuerdo/ni en desacuerdo”. Por favor, trata de usar esta categoría cuando verdaderamente no tengas una opinión clara.

1 = Estoy totalmente de acuerdo
2 = Estoy de acuerdo
3 = Ni estoy de acuerdo/ni en desacuerdo
4 = No estoy de acuerdo
5 = Estoy totalmente en desacuerdo

Sentí que los profesionales me escuchaban	1	2	3	4	5
Sentí que podía expresarme emocionalmente delante de los profesionales	1	2	3	4	5
Los profesionales fueron siempre respetuosos hacia mí y hacia mi familia	1	2	3	4	5
Me / Nos dieron bastante información para ayudarnos con las decisiones que tuvimos que tomar					

Los profesionales fueron sensibles en el uso del lenguaje	1 2 3 4 5
Aunque perdí mi bebé fui tratada como una madre	1 2 3 4 5
Me sentí emocionalmente apoyada por los/as médicos (ej. ginecólogos, obstetras)	1 2 3 4 5
Me sentí emocionalmente apoyada por los/as enfermeras y matronas	1 2 3 4 5
Sentí que podría hacerles preguntas si quería	1 2 3 4 5
Me / Nos ayudaron bastante para guardar recuerdos físicos del bebé/s, como una fotografía, la pulsera de identificación, etc.	1 2 3 4 5
Algunos de los profesionales me trataron bien y otros mal	1 2 3 4 5
Las enfermeras/matronas parecían saber cómo tratar con casos de pérdida	1 2 3 4 5
Los médicos no parecían saber cómo tratar con casos de pérdida	1 2 3 4 5

63 ¿Cuáles de los siguientes estudios patológicos o pruebas médicas te ofrecieron (luego te preguntaremos sobre cuales se realizaron)?
Marca todas las opciones que sean necesarias

Ninguno 1
Autopsia general/ necropsia 2
Autopsia de la placenta 3
Biopsia 4
Estudio fenotípico (estudio genético) 6
Otro, especificar por favor 7

64 Por favor, indícanos quién te/os explicó las posibilidades de realizar estudios patológicos o pruebas médicas.
Marca todas las opciones que sean necesarias

Nadie 1
Un/a médico 2
Un/a patólogo 3
Un/a matrona o enfermera/o 4
Otro, especificar por favor 5

65 Por favor, indícanos en qué momento te/os hablaron de la posibilidad de realizar estudios patológicos o pruebas médicas.
Marca todas las opciones que sean necesarias

Antes del parto 1
Durante el parto 2
Después del parto 3

66 Te hemos preguntado qué estudios patológicos o pruebas médicas te ofrecieron, ahora nos gustaría saber cuáles se realizaron.
Marca todas las opciones que sean necesarias

Autopsia general/ necropsia 1
Autopsia de la placenta .... 2 → P68
Biopsia .... 3 → P68
Estudio fenotípico (estudio genético) .... 4 → P75
No, ninguna prueba, ni patológica ni médica .. 5 → P75

Si se realizó una autopsia…
67 ¿Tu o tu pareja firmasteis un consentimiento oficial para la autopsia?

Sí 1
No 2
No sé / no recuerdo 3

Si se realizó una autopsia o biopsia…
68 ¿Cuánto tiempo tardaron en llegar los resultados de la autopsia o biopsia?
Si hubo autopsia y biopsia, contesta sobre la autopsia

Menos de 1 mes 1
1-3 meses 2
3-6 meses 3
Más de 6 meses 4
Aún no han llegado .... 5 → P75

Si ya has recibido los resultados de la autopsia o biopsia.
69 ¿Cómo te llegaron los resultados?

Por correo ordinario 1
Con cita en el hospital/durante una revisión 2
Por correo electrónico 3
Reclamando/visita de reclamación 4
Otro, especificar por favor 5

70 Por favor, indícanos quién te/os explicó los resultados de la autopsia o biopsia.
Marca todas las opciones que sean necesarias

Nadie 1
Un/a médico 2
Un/a patólogo 3
Un/a matrona o enfermera/o 4
Otro, especificar por favor 6

71 Con respecto a la explicación de los resultados de la autopsia o biopsia, indica tu grado de acuerdo con la siguiente frase:

1 = Estoy totalmente de acuerdo
2 = Estoy de acuerdo
3 = Ni estoy de acuerdo/ni en desacuerdo
4 = No estoy de acuerdo
5 = Estoy totalmente en desacuerdo

Me explicaron de una manera clara y entendible los resultados de la autopsia o biopsia 1 2 3 4 5

Si ya has recibido los resultados de la autopsia o biopsia.
72 ¿Proporcionó la autopsia o biopsia una causa de muerte?

Sí 1
Sí, pero no definitiva 2
No 3

Si no fuera una obligación legal / Si se realizó una autopsia (no biopsia)…
73 ¿Cuál de las siguientes opciones describe mejor tus sentimientos actuales sobre la decisión de autorizar la autopsia?

Fue una buena decisión 1
Estoy indecisa, no sé si fue una buena decisión 2
Ojalá no lo hubiera hecho 3

Si se realizó una autopsia o biopsia…
74 ¿Hay algo relacionado con la autopsia, biopsia u otra prueba médica que no hemos preguntado que le gustaría contar?
Puedes contárnoslo en el espacio de abajo

75 ¿Quién te/os explicó el procedimiento y opciones para la disposición del cuerpo?
Marca todas las opciones que sean necesarias

Nadie	1
Un/a médico	2
Un/a matrona/ o enfermera/o	3
Alguien de la funeraria	4
Trabajador o asistenta social	5
Celador	6
Administrativo	7
Se encargo pareja/un familiar	8
Otro, especificar por favor	9

76 ¿Cuál de las siguientes opciones describe mejor el procedimiento del funeral o la disposición de su cuerpo/s?

Donamos su cuerpo/s a la investigación	1	→ P78
Entierro particular	2	→ P78
Cremación particular mediante funeraria, recuperamos las cenizas	3	→ P78
Cremación en el hospital, no recuperamos las cenizas	4	
No nos devolvieron el cuerpo/s al ser una pérdida temprana	5	→ P78
Nos dijeron que si hubo autopsia no podemos recuperar el cuerpo/s	6	→ P78
Entierro (fosa común) mediante el hospital	7	
Cremación particular sin recuperar las cenizas	8	
No sé/ no había opciones	9	
Otro, especificar por favor	10	→ P78

77 Nos has indicado que elegiste cremar en el hospital sin poder recuperar las cenizas, indícanos abajo porqué elegiste esta opción:

Falta de información/mala comunicación	1
Decisión rápida/apresurada/en estado de shock	2
Parecía mejor opción/fue mejor opción	3
No había otra opción/dijeron que era lo habitual	4
Era el protocolo	5
Madre no participó en la decisión	6
Pensaba que íban a devolver las cenizas	7

78 Pensando en los procedimientos de cuidados en el hospital, marca el número que mejor indique el grado de acuerdo o desacuerdo actualmente con cada una de las siguientes frases. Si no estás segura, usa la categoría "Ni estoy de acuerdo/ni en desacuerdo". Por favor, trata de usar esta categoría cuando verdaderamente no tengas una opinión clara.

1 = Estoy totalmente de acuerdo
2 = Estoy de acuerdo
3 = Ni estoy de acuerdo/ni en desacuerdo
4 = No estoy de acuerdo
5 = Estoy totalmente en desacuerdo

Me explicaron de una manera clara y entendible el proceso del parto en casos de pérdida.	1	2	3	4	5
Me/nos presentaron los informes oficiales relacionados con la muerte en un momento adecuado (certificados de defunción, autopsia, nacimiento, boletín estadístico, etc.)	1	2	3	4	5
La habitación donde estaba alojada era un lugar tranquilo	1	2	3	4	5
Todo el personal de la planta estuvo al tanto de mi situación	1	2	3	4	5
En general me/nos informaron bien sobre todos los pasos y trámites durante la estancia hospitalaria	1	2	3	4	5
Los/as médicos me parecieron competentes en su trabajo	1	2	3	4	5
Los/as enfermeras/matronas me parecieron competentes en su trabajo	1	2	3	4	5
Los/as médicos, matronas y enfermeras parecían trabajar bien en equipo	1	2	3	4	5
Había un/una profesional que me/nos guió durante el proceso	1	2	3	4	5
Sentí que tuve control sobre las decisiones relacionadas con los aspectos médicos (ej. el parto, sedantes)	1	2	3	4	5
Sentí que tuve control sobre las decisiones relacionadas con los aspectos de ritual (ej. cómo ver/ sostener)	1	2	3	4	5

79 Por favor, usando la escala, indica la cantidad de información (verbal o escrita) que recibiste durante la estancia hospitalaria, respecto a los siguientes temas:

1 = Nada
2 = Poca
3 = Bastante
4 = Mucha

Información sobre la posibilidad de guardar recuerdos físicos del bebé/s, como una fotografía, la pulsera de identificación, etc.	1	2	3	4
Información sobre el proceso de duelo y su desarrollo normal	1	2	3	4
Información sobre dónde podría encontrar información sobre el duelo perinatal/gestacional (ej. páginas web, libros)	1	2	3	4
Información sobre auto-cuidados en el puerperio y para después del alta (ej. con la lactancia)	1	2	3	4
Información acerca de la disposición del cuerpo (ej. el manejo del funeral / cremación, etc.)	1	2	3	4
Información acerca de la autopsia y otras pruebas médicas	1	2	3	4

80 Pensando en la estancia hospitalaria, ¿qué fue lo que más te ayudó (de lo que alguien hizo o dijo)?

81 Pensando en la estancia hospitalaria, ¿qué fue lo que menos te ayudó (de lo que alguien hizo o dijo)?

82 Cada una de estas afirmaciones representan los pensamientos y sentimientos de algunas personas que han tenido una pérdida similar a la tuya. En estas afirmaciones no hay respuestas correctas ni incorrectas. Marca el número que mejor indique el grado de acuerdo o desacuerdo actualmente con cada una de ellas. Si no estás segura, usa la categoría "Ni estoy de acuerdo/ni en desacuerdo". Por favor, trata de usar esta categoría cuando verdaderamente no tengas una opinión clara.

1 = Estoy totalmente de acuerdo
2 = Estoy de acuerdo
3 = Ni estoy de acuerdo/ni en desacuerdo
4 = No estoy de acuerdo
5 = Estoy totalmente en desacuerdo

Me siento deprimida	1	2	3	4	5
Se me hace difícil llevarme bien con ciertas personas	1	2	3	4	5
Siento un vacío interior	1	2	3	4	5

No puedo seguir el ritmo de mis

actividades cotidianas	1	2	3	4	5
Siento la necesidad de hablar de mi bebé	1	2	3	4	5
Estoy en duelo por mi bebé	1	2	3	4	5
Estoy asustada	1	2	3	4	5
He pensado en suicidarme desde que perdí a mi bebé	1	2	3	4	5
Tomo pastillas para los nervios	1	2	3	4	5
Echo mucho de menos a mi bebé	1	2	3	4	5
Siento que me he adaptado bien a la pérdida	1	2	3	4	5
Es doloroso recordar la pérdida de mi bebé	1	2	3	4	5
Me altero cuando pienso en mi bebé	1	2	3	4	5
Lloro cuando pienso en mi bebé	1	2	3	4	5
Me siento culpable cuando pienso en mi bebé.	1	2	3	4	5
Me siento físicamente enferma cuando pienso en mi bebé	1	2	3	4	5
Me siento desprotegida en un entorno hostil desde que mi bebé murió	1	2	3	4	5
Trato de reírme pero ya nada me hace gracia	1	2	3	4	5
El tiempo pasa muy lentamente desde que murió mi bebé	1	2	3	4	5
Lo mejor de mí murió con mi bebé	1	2	3	4	5
He decepcionado a personas desde que murió mi bebé	1	2	3	4	5
Siento que no valgo nada desde que mi bebé murió	1	2	3	4	5
Me culpo por la muerte de mi bebé	1	2	3	4	5
Me enfado más de lo que debiera con mis amigos y familiares	1	2	3	4	5
Algunas veces siento que necesito ayuda profesional para rehacer mi vida	1	2	3	4	5
Me siento como muerta en vida desde que murió mi bebé	1	2	3	4	5
Me siento muy sola desde que mi bebé murió	1	2	3	4	5
Me siento apartada y aislada incluso cuando estoy con mis amigos	1	2	3	4	5
Siento que es mejor no querer a nadie	1	2	3	4	5
Se me hace difícil tomar decisiones desde que murió mi bebé	1	2	3	4	5
Me preocupa cómo será mi futuro	1	2	3	4	5
Siento que mi dolor por la pérdida de mi bebé es invisible ante la sociedad	1	2	3	4	5
Me siento feliz por el simple hecho de estar viva	1	2	3	4	5

Si las semanas de gestación son 22 o más

83 ¿Antes del alta te dieron el Boletín Estadístico de Parto?

El Boletín Estadístico de Parto es un documento del Registro Civil con muchas casillas que tiene los datos sobre los padres y del parto, como el sexo, las semanas de gestación. Es un documento que el ginecólogo debería darte rellenado y firmado.

No 1
Sí, completamente rellenado 2
Sí, parcialmente rellenado 3
Sí, pero no estaba rellenado 4
No sé / no recuerdo 5

Si recibieron el BEP

84 ¿Has llevado (u otra persona) el Boletín Estadístico de Parto al Registro Civil de tu ciudad o municipio?

Sí, lo llevé/llevamos 1
Sí, se encargó la funeraria 2
Todavía no, estoy en ello 3
No, no me acordé/no nos acordamos llevarlo 4
No, no sabía/sabíamos que había que llevar un boletín estadístico de parto para una pérdida perinatal 5
No sé / no recuerdo 6

85 Después de que te dieran el alta, ¿cuál de las siguientes opciones describe mejor la forma en que te hicieron el seguimiento?

Marca todas las opciones que sean necesarias

No tuve ningún seguimiento después del alta/ aún no porque es pronto 1
Fui a una revisión en el mismo hospital 2
La matrona del centro salud me visitó en casa 3
Fui a una cita con la matrona en el centro salud 4
Fui a una cita con el/la médico en el centro salud 5
Me siguieron desde Salud Mental 6
Fui al ginecólogo a las 6 semanas 7
Fui al ginecólogo a las 1-3 semanas 8
Revisión con ginecólogo privado 9
Seguimiento de especialidades/diagnósticos 10
Urgencias por complicaciones/restos 11
Revisión periódica/ continua 12
Otro, especificar por favor 13

86 Pensando en cómo te sientes actualmente, ¿cuál de las siguientes opciones describe mejor cómo estás sobrellevando la muerte de tu bebé/s?

Muy mal (todos los días me parecen malos) 1
Mal pero no del todo (más días malos que buenos) 2
Ni mal ni bien (los días malos y buenos me parecen iguales) 3
Bastante bien (más días buenos que malos) 4
Muy bien (la mayoría de los días son buenos con alguno malo a vez en cuando) 5
No lo sé 6

87 ¿Has recibido apoyo psicológico / terapia, respecto a la pérdida, en alguna de las siguientes fases o momentos?

Marca todas las opciones que sean necesarias

No 1
Después del alta 2
Durante un embarazo posterior 3
Ambos, después del alta y durante un embarazo posterior 4

88 ¿Dónde acudiste para tal apoyo psicológico / terapia?

Marca todas las opciones que sean necesarias

Psicólogos/psiquiatras de salud mental (público) 1
Enfermera de la unidad de salud mental (público) 2
Un psicólogo/psiquiatra privado general 3
Un psicólogo/psiquiatra privado especialista en duelo 4
Un psicólogo/psiquiatra privado especialista en duelo gestacional / neonatal 5
Asociación de apoyo al duelo general 6
Asociación/grupo de apoyo padres 7
Terapia alternativa/natural 8
Psicólogos/psiquiatras de salud mental (público) especialista 9
Otro, especificar por favor 10

Si sigue casada/pareja de hecho

89 ¿Tu pareja ha recibido apoyo psicológico / terapia, respecto a la pérdida, en alguna de las siguientes fases o momentos?
Marca todas las opciones que sean necesarias

No 1
Después del alta 1
Durante un embarazo posterior 2
Ambos después del alta y durante un embarazo posterior 4

90 ¿Dónde acudió para tal apoyo psicológico/ terapia?
Marca todas las opciones que sean necesarias

Psicólogos/psiquiatras de salud mental (público) 1
Enfermera de la unidad de salud mental (público) ....
Un psicólogo/psiquiatra privado general 2
Un psicólogo/psiquiatra privado especialista en duelo . 3
Un psicólogo/psiquiatra privado especialista en duelo gestacional / neonatal 4
Asociación de apoyo al duelo general 6
Asociación/grupo de apoyo padres 7
Terapia alternativa/natural 8
Psicólogos/psiquiatras de salud mental (público) especialista 9
Otro, especificar por favor 10

Filtrar, excluir embarazos de múltiples

91 ¿Si hubieras tenido la posibilidad de donar tu leche a un banco de leche lo hubieras hecho?

Sí, definitivamente 1
Sí, probablemente 2
No sé, no estoy segura 3
No, definitivamente 4

92 Por favor, indícanos si crees que hubo alguna negligencia médica en tu caso.

No 1
Sí, fue denunciado 2
Sí, no fue denunciado 3
No sé, no estoy segura 4

93 ¿Has buscado información sobre la muerte perinatal en internet en algún momento?
Marca todas las opciones que sean necesarias

No, nunca 1
Sí, durante la estancia hospitalaria 2
Sí, después del alta 3

94 ¿Has tenido contacto con algún grupo o asociación de apoyo o con otras madres/padres que han experimentado una muerte perinatal?
Marca todas las opciones que sean necesarias

No 1
Sí, he estado en contacto con una asociación o un grupo de apoyo por teléfono o e-mail 2
Sí, he visitado o participado en un grupo de apoyo online 3
Sí, he visitado o participado en un grupo de apoyo presencial 4
Sí, he estado en contacto con otros madres/padres pero no en un grupo de apoyo formal 5
Sí, he asistido a un evento de conmemoración 6

95 ¿Cómo valorarías el nivel de apoyo que recibiste de las siguientes personas?

Nada de apoyo = 1
Un poco de apoyo = 2
Bastante apoyo = 3
Mucho apoyo = 4
No relevante/no les conozco = 5

1) Enfermeras/matronas	1	2	3	4	5
2) Médicos	1	2	3	4	5
3) Pareja	1	2	3	4	5
4) Familia cercana	1	2	3	4	5
5) Familia en general	1	2	3	4	5
6) Amigos cercanos	1	2	3	4	5
7) Amigos en general	1	2	3	4	5
8) Un psicólogo/psiquiatra	1	2	3	4	5
9) Otras madres/padres que han tenido una pérdida	1	2	3	4	5
10) Un grupo de apoyo online	1	2	3	4	5
11) Un grupo de apoyo presencial	1	2	3	4	5
12) Compañeros de trabajo	1	2	3	4	5
13) Un asociación de apoyo	1	2	3	4	5

96 Pensando en tus sentimientos generales sobre los cuidados en el hospital, marca el número que mejor indique el grado de acuerdo o desacuerdo actualmente con cada una de las siguientes frases. Si no estás segura, usa la categoría “Ni estoy de acuerdo/ni en desacuerdo”. Por favor, trata de usar esta categoría cuando verdaderamente no tengas una opinión clara.

1 = Estoy totalmente de acuerdo
2 = Estoy de acuerdo
3 = Ni estoy de acuerdo/ni en desacuerdo
4 = No estoy de acuerdo
5 = Estoy totalmente en desacuerdo

A pesar de las circunstancias me siento satisfecha con los cuidados que recibí en el hospital	1	2	3	4	5
Recomendaría este hospital a otras madres / parejas	1	2	3	4	5

97 ¿Cómo te defines en materia religiosa?

Católica 1
Evangélica o protestante 2
Judía 3
Musulmana 4
Mormón 5
Ortodoxa 6
Budista 7
Testigo de Jehová 8
No creyente / agnóstica 9
Atea 10
Estoy en un momento de dudar de mis creencias 11
Otra 12

Si está en pareja (aparece por filtro)

98 ¿Cuál de las siguientes opciones describe mejor el nivel de estudios de tu pareja?

Fue menos de 5 años a la escuela 1
Fue a la escuela 5 años o más pero sin completar EGB, ESO o Bachillerato elemental 2
Bachiller elemental, EGB o ESO completa (Graduado escolar) 3
Bachiller superior, BUP, Bachiller LOGSE, COU, PREU 4
FPI, FP grado medio, Oficialía industrial o equivalente 5
FPII, FP superior, Maestría industrial o equivalente 6
Diplomatura, Arquitectura o Ingeniería técnica; 3 cursos aprobados de Arquitectura, Ingeniería o equivalente 7
Licenciatura o equivalente 8
Master o doctorado 9

Si está en pareja (aparece por filtro)

99 ¿Cuál de las siguientes opciones describe mejor la ocupación de tu pareja?

Profesional, técnico 1
Directivo de la administración pública y de empresas 2
Personal administrativo 3
Comerciante y vendedor 4
Personal de servicios 5
Agricultor, ganadero, arboricultor, pescador y cazador 6
Trabajador de la producción, conductores de equipos de transportes y peones (no agrarios 7
De transportes y peones (no agrarios) 8
Profesional de las fuerzas armadas 9
Estudiante 10
Persona dedicada a las labores de su hogar 11
Jubilado, retirado, pensionista y rentista 12
Persona que no puede ser clasificada 13

100 ¿A qué clase social dirías que perteneces?

Alta 1
Media-alta 2
Media 3
Media-baja 4
Baja 5

101 ¿Cuál de las siguientes opciones describe mejor donde vives?

En una capital de provincia 1
En los suburbios/periferia de una capital de provincia 2
En una ciudad pequeña o un pueblo grande 3
En un pueblo 4
En una aldea 5
En una granja o una casa en el campo 6

102 Actualmente, entre todos los miembros del hogar y por todos los conceptos, ¿en qué franja estarían los ingresos netos de tu hogar al mes?

Menos o igual a 300 € *1*
De 301 a 600 € *2*
De 601 a 900 € *3*
De 901 a 1.200 € *4*
De 1.201 a 1.800 € *5*
De 1.801 a 2.400 € *6*
De 2.401 a 3.000 € *7*
De 3.000 a 4.500 € *8*
De 4.501 a 6.000 € *9*
Más de 6.000 € *10*

103 ¿Estarías interesada en ayudarnos con otras investigaciones que realicemos? También te pasaremos los resultados de la investigación en cuanto se publiquen.

Sí, me gustaría participar 1
Tal vez, pero me gustaría recibir más información antes de comprometerme 2
No, gracias .... 3 → P105

104 Nos has indicado que te gustaría participar en otras investigaciones o recibir más información, por favor deja tu nombre, teléfono y e-mail y estaremos en contacto. Recuerda que todos tus datos personales son estrictamente confidenciales y tu nombre nunca aparecerá relacionado a los resultados de la investigación.

Nombre	
Teléfono	
E-mail	

Si contesta que no quiere participar en otras investigaciones

105 ¿Estarías interesada en recibir noticias sobre los resultados de esta encuesta?

Sí 1
No .... 3 → P107

Si contesta que no quiere participar en otras investigaciones

106 Nos has indicado que te gustaría recibir los resultados de la investigación, por favor deja tu nombre y e-mail, estaremos en contacto. Recuerda que todos tus datos personales son estrictamente confidenciales y tu nombre nunca aparecerá relacionado a los resultados de la investigación.

Nombre	
E-mail	

107 Si hay algo sobre la encuesta que te gustaría comentarnos que te parece importante o que hemos olvidado preguntar puedes contárnoslo en el espacio de abajo.

Muchas gracias por tu ayuda, nos comprometemos a usar la información que nos has proporcionado para promover mejoras en los cuidados que reciben los padres después de una muerte perinatal.

AYÚDANOS CONTACTAR CON OTRAS MADRES

Es muy importante para esta investigación que contactemos con el mayor número de madres posible. Si conoces a otra madre o un familiar de otra madre que ha sufrido una pérdida gestacional entre la semana 16 y el parto, te agradeceríamos que copiases este enlace y que se lo enviases por correo electrónico o en el caso de no saber su correo electrónico que se lo comunicases por otro medio.

Enlace para mandar la encuesta a otras madres:

XXXX

INFORMACIÓN ACERCA DE LA ENCUESTA:

Si te gustaría recibir más información acerca del cuestionario puedes contactar con Paul Cassidy por e-mail: paulcassidy@umamanita.es

INFORMACIÓN SOBRE EL DUELO PERINATAL Y APOYO

Si te gustaría obtener más información acerca del duelo perinatal y apoyo te invitamos a visitar nuestra página web o página de Facebook:

http://www.umamanita.es
https://www.facebook.com/uma.manita?fref=ts

Para información acerca de apoyo grupal, pincha aquí:

Grupo de apoyo a la pérdida

También podemos recomendar las siguientes asociaciones que apoyan a madres y padres después de la pérdida perinatal y gestacional:

Petits am llum
http://www.petitsambllum.org/

Superando un aborto (SUA)
http://superandounaborto.foroactivo.com/
