# Supplementary material for: Care quality following intrauterine death in Spanish hospitals: results from an online survey
Source: BMC Pregnancy Childbirth. 2018 Jan 10;18:22. doi: 10.1186/s12884-017-1630-z (PMC5763533; doi:10.1186/s12884-017-1630-z)
Supplement: Supplementary file 2 — Questionnaire (English). (PDF 394 kb) [file 12884_2017_1630_MOESM2_ESM.pdf]

ADDITIONAL FILE 3.
QUESTIONNAIRE (ENGLISH)

ARTICLE TITLE
Care quality following intrauterine death in Spanish hospitals: Results from an online survey

AUTHOR
Paul Richard Cassidy BBS MPhil

Doctoral student
Universidad Complutense de Madrid
Facultad de Ciencia Política y Sociología
Somosaguas, Pozuelo de Alarcon
Madrid, 28223
Email: pcassidy@ucm.es

Researcher
Umamanita (Stillbirth Charity)
C/ Hierbabuena 15, Esc B, 4 Izq
Madrid, 28039
Email: paulcassidy@umamanita.es

SURVEY FOR MOTHERS ON THEIR EXPERIENCES IN THE SPANISH HEALTHCARE SYSTEM FOLLOWING AN INTRAUTERINE FETAL DEATH

ENGLISH TRANSLATED VERSION - NOT TESTED

ABOUT THE SURVEY:

- The objective of this study is to better understand the experiences of parents in the hospital during and after an intrauterine death between 16 weeks and up to and including during labour.
- The questionnaire has been developed with the support of Umamanita, a charity that supports parents following perinatal death.

WHO SHOULD FILL IN THE QUESTIONNAIRE?

- Although we understand that the number of weeks gestation at which a death occurs is often not important for parents, for reasons to do with care processes, this questionnaire is only for mothers who have experienced an intrauterine death from 16 weeks gestation and up to and including during labour. If your baby was born alive (neonatal death) the questionnaire is not applicable. The questionnaire is designed for the following classes of death: spontaneous intrauterine death (stillbirth), medical termination of pregnancy due to threat to the mother's life or incompatibility with life of the fetus and selective reduction in multiple pregnancies.
- Although the questionnaire is only applicable for mothers that does not preclude the possibility that your partner or other family member might be able to help you fill it in, we understand that in many instances they will handle paperwork or arrangements for disposition, or have clearer memories of certain aspects of care.
- If you have had more than one pregnancy loss, please answer the questionnaire only in relation to the last loss.
- If you have experienced multiple losses in the same pregnancy (e.g. twins or triplets), you should only answer the questionnaire once.

IMPORTANT! PLEASE READ BEFORE FILLING IN THE QUESTIONNAIRE:

- We are conscious that the questionnaire deals with an emotionally difficult subject and we are very grateful for your participation. Our commitment is to use the information that you provide to promote care improvement.
- It is important to remember that there are no right and wrong answers, only your personal opinion, therefore it is very important for the reliability of the questionnaire that your answers reflect only your personal experiences.
- If there is a question that you don't understand or that you think you cannot answer accurately, don't worry, leave it blank and continue to the next question.
- The questionnaire should take about 45 minutes to complete and it is better that you complete it in one sitting, however, if you have to stop, don't worry, just leave the questionnaire open in your internet browser.
- The questionnaire is anonymous. Remember that all your answers will only be used for statistical purposes and never individually. We guarantee absolute confidentiality in accordance with the Spanish Data (Personal) Protection Law 15/99.
- If you have any doubts or questions about the research, please get in touch with Paul Cassidy by email: investigación@umamanita.es

Before we start we'd like to ask you some questions to help us classify your answers.

1 Please select the province where you currently live?

Drop down list

2 Please select the province where you lived at the time of the loss?

Drop down list

3A Was the hospital you attended public or private?

- *Public hospital* .... 1 → P3C
- *Private hospital* .... 2 → P3C
- *First private and then public* 3
- *First public and then private* 4

If the respondent was transferred to another hospital

3B When were you transferred to the second hospital

- *After the diagnosis* 1
- *After the birth* 2
- *Other, please specify* 3

3C What is the name of the hospital where you were admitted? If you were admitted to more than one hospital, state the hospital where you spent more time (nights spent in the hospital)

Open-ended text field

4 How old are you? Number field

5 What is your nationality? Drop down list

6 Which of the following options best describes your education level?

- *Unable to read or write (help with answering the questionnaire)* 1
- *5 or less years of schooling* 2
- *5 or more years of schooling but without completing the lower second level exams* 3
- *Lower second level* 4
- *Upper second level* 5
- *Professional/vocational level I* 6
- *Professional/vocational level II* 7
- *Third level diploma* 8
- *Third level degree* 9
- *Master or doctorate* 10

7 Which of the following options best describes your occupation?

- *Professional or technical* 1
- *Executive/director of a public body or a company* 2
- *Administrative worker* 3
- *Merchant or salesperson* 4
- *Service sector worker* 5
- *Agricultural or fisheries sector worker* 6
- *Production worker, transport equipment drivers and labourers (non-agricultural)* 7
- *Member of the armed forces* 8
- *Student* 9
- *Home worker* 10
- *Pensioner, retired or in receipt of an annuity* 11
- *Person who can't be classified* 12

8 What is your current marital status?

- *Married, civil union or cohabiting with a partner* 1
- *Single* 2
- *Widow* 3
- *Separated* 4
- *Divorced* 5
- *Divorced and newly married, civil union or cohabiting with a partner* 6

9 What was your marital status at the time of the loss?

- *Married, civil union or cohabiting with a partner* 1
- *Single* 2
- *Widow* 3
- *Separated* 4
- *Divorced* 5

10 What type of pregnancy did you have?

- *Single* 1
- *Twin* 2
- *Triplets* 3
- *Quadruplets or more* 4

11 Which of the following options best describes the type of loss you had?

- *Spontaneous intrauterine death* 1
- *Medical termination due to problems with the baby's health* 2
- *Medical termination due to a threat to the mother's health* 3
- *Selective reduction of a multiple pregnancy* 4
- *Neonatal* 5
- *Death during labour* 6
- *Other, please specify* 7

Open text (short)

12 How many weeks pregnant were you when your baby died?

- *Between 16 and 19 weeks* 1
- *Between 20 and 21 weeks* 2
- *Between 22 and 25 weeks* 3
- *Between 26 and 29 weeks* 4
- *Between 30 and 33 weeks* 5
- *Between 34 and 36 weeks* 6
- *Between 37 and 41 weeks* 7
- *Between 42 y birth (not during the birth)* 8
- *During the birth/labour* 9

13 How many babies did you lose?

Boys Number field *Girls* Number field

14A In what month and year did you lose your baby/s?

Month Drop down list *Year* Drop down list

14B When did the death occur?

- *During the last six weeks* *1*
- *During the last 3 months (90 days)* *2*
- *Between 4 and 6 months ago* *3*
- *Between 7 and 12 months ago* *4*
- *More than a year ago (12 months)* *5*

15 Did you have fertilization treatment/assistance (IVF) during the conception?

- *Yes* 1
- *No* 2

16 Had you ever had any of the following types of pregnancy loss or perinatal death prior to the last loss/death?
Please tick all that apply

- *No* ... 1
- *Early miscarriage (up to 12 weeks gestation)* ... 2
- *Late miscarriage (between 13 and 19 weeks gestation)* ... 3
- *Spontaneous intrauterine death after 20 weeks gestation* ... 4
- *Selective reduction of a multiple pregnancy* ... 5
- *Medical termination due to problems with the baby's health* ... 6
- *Medical termination due to a threat to the mother's health* ... 7
- *Neonatal death up to 28 after birth* ... 8
- *Death of a child after 28 days* ... 9
- *Other, please specify* ... 10

Open text (short)

If the loss/death occurred more than three months ago...

17 This questionnaire is about your last loss between 16 weeks and birth. Since that loss have you had an earlier loss (up to 15 weeks)?

- *No* ... 0
- *Yes, I've had one early loss* ... 1
- *Yes, I've had more than one early loss* ... 2

18 Did you have any children before this loss?

- *Yes* ... 1
- *No* ... 2

If the loss/death occurred more than six weeks ago...

19 Are you pregnant at the moment or have you had any children born alive since your last loss at 16 weeks of more?
Tick all that apply

- *I'm pregnant at the moment* ... 1
- *Yes, I've had a child/children born alive* ... 2
- *No* ... 3

20 Did you know the gender of your baby before you received the diagnosis that your baby had died?

- *Yes* ... 1
- *No* ... 2 → P22

21 If you knew your baby's gender before the diagnosis, had you given him/her a name?

- *Yes* ... 1
- *No* ... 2

22 Did you have any of the following complications during the pregnancy?
Tick all that apply

- *I didn't have any complications during the pregnancy* 1
- *Gestational diabetes: excess sugar in the blood* ... 2
- *Mellitus diabetes type I: insulin dependent* ... 3
- *Hyperemesis gravidarum (Hyperemesis gravidarum, HG): Nausea and intense and persistent vomiting during pregnancy, more extreme than "morning sickness"* ... 4
- *Hypertension (pregnancy-related): Hypertension that begins after 20 weeks of pregnancy and disappears after childbirth* ... 5
- *Anemia: Lower level of healthy red blood cells than normal* ... 6
- *Placental abruption: The placenta separates from the uterine wall before delivery, which may mean that the baby is not getting enough oxygen* ... 7
- *Placenta previa: The placenta covers the entire opening of the cervix inside the uterus or part of it* ... 8
- *Intrauterine growth restriction* ... 9
- *Reduced amniotic fluid (oligohydramnios)* ... 10
- *Chorioamnionitis (infection of the placental membranes and amniotic fluid)* ... 11
- *Maternal thyroid problems* ... 12
- *Maternal obesity or significant weight problems* ... 13
- *Threat of premature birth* ... 14
- *Premature rupture of membranes* ... 15
- *Incompetent cervix* ... 16
- *Threat of misscarriage (up to 20 weeks)* ... 17
- *Vaginal bleeding/ spotting* ... 18
- *Cholestasis (pregnancy)* ... 19
- *Excess amniotic fluid* ... 20
- *Other, please specify* ... 21

Open text (short)

23 Where were you when the told you that your baby had died or that the diagnosis was not very good?

- *Emergency room* ... 1
- *Doctor's office* ... 2
- *Family room* ... 3
- *Surgical reanimation* ... 4
- *Neonatal unit / neonatal intensive care* ... 5
- *Intensive care* ... 6
- *Labour room* ... 7
- *Ultrasound room* ... 8
- *Health centre* ... 9
- *Room in the hospital* ... 10
- *Surgery* ... 11
- *Dilation room* ... 12
- *By telephone* ... 13
- *At home* ... 14
- *Other, please specify* ... 15

Open text (short)

24 Who communicated the bad news to you?

- *A doctor* ... 1
- *A midwife or a nurse* ... 2
- *My partner or other family member* ... 3 → P26
- *Other, please specify* ... 4

Open text (short)

If a doctor, midwife or nurse communicated the bad news...

25 Were you accompanied by anyone or were you alone when they told you your baby had died or that the diagnosis was not good?

- *Yes, by my partner* ... 1
- *Yes, by a family member or a friend* ... 2
- *No, I was alone* ... 3 → P27

If the mother was accompanied during the diagnosis

26 Did they offer you a private place to be with your partner or family after they gave you the diagnosis?

Yes 1
No 2

27 Now, we'd like you to think about your interactions with the health professionals around the time they told you your baby had died or that the diagnosis was not good.

Choose the number which best reflects your current level of agreement or disagreement with each of the following statements. If you are not sure, use the category 'neither agree nor disagree'. Please only use this category when you definitely don't have a clear opinion.

1 = I completely agree
2 = I agree
3 = I neither agree nor disagree
4 = I disagree
5 = I completely disagree

When I arrived at the hospital or health centre I was treated with priority	1	2	3	4	5
I could tell by the reaction of the health professional that the news was bad	1	2	3	4	5
I could tell by looking at the monitor that the news was bad	1	2	3	4	5
The doctor took a long time to come and see me	1	2	3	4	5
I received a clear explanation of the diagnosis in easy to understand language	1	2	3	4	5
I had an opportunity to ask questions about the diagnosis	1	2	3	4	5
The person who gave me the bad news was empathetic and understanding	1	2	3	4	5
I felt accompanied by the health professionals in the time immediately after receiving the bad news	1	2	3	4	5

28 Which of the following best describes your accommodation?

Private room (not shared) 1
Shared room with an obstetric/ maternity . 2 → P30
Shared room with a non-obstetric/ maternity patient 3
General ward 4
Emergency ward 5
Dilation room/ delivery room 6
First a shared room and then private 7
Room shared with another mother who had had a pregnancy loss 8
Not admitted for an overnight 9
Other, please specify 10
Open text (short)

29 Could you hear babies in the maternity ward crying from your room?

No 1
Somewhat 2
Yes 3

30 What type of birth did you have?

Eutocic or spontaneous labour (vaginal delivery that starts spontaneously, i.e., without medication and ends spontaneously, i.e., it is not necessary to perform surgical procedures to facilitate the delivery of the baby) 1
Dystocia (vaginal delivery in which physical manoeuvres or surgery is used to completion the delivery, that is, instruments are needed to facilitate the delivery of the baby, usually suction, spatulas or forceps) 2
Stimulated-induced labour (vaginal delivery in which it is necessary to induce contractions of labour by medication: prostaglandins and / or oxytocin) 3
Stimulated-induced and dystocia labour (vaginal childbirth in which contractions of labour are induced by prostaglandins and / or oxytocin and the birth ends with surgical manoeuvres to facilitate delivery of the baby, usually suction, spatulas or forceps). 4
Planned cesarean 5
Emergency cesarean 6
Cesarean due to failed induction 7
Other, please specify 8
Open text (short)

31 Were you accompanied by anyone during the birth?

Yes, by my partner .... 1 → P33
Yes, by a family member or friend .... 2 → P33
No, I was alone 3

32 You indicated that you were alone during the birth, could you tell us why?

I wanted it that way 1
There wasn't anyone to be with me 2
My partner didn't want to come in 3
They didn't let my partner / family member / friend enter 4
Because I was under general anaesthetic 5
Other, please specify 6
Open text (short)

33 Apart from the loss, was there any complication during the birth?

Yes 1
No 2

34 How many nights did you spend in the hospital?

Less than 1 day, I didn't spend the night 1
1-2 nights 2
3-4 nights 3
5-7 nights 4
8 nights - 2 weeks 5
More than 2 weeks 6

35 Were you given tranquilizers or sedatives at any stage during the hospital stay? (medication to sedate or tranquilize NOT medication for pain or anaesthetics related to labour or sleeping pills)
Tick all that apply

No .... 1 → P43
Yes, after the diagnosis or during the initial stages of the labour 2
Yes, during the labour (either just before or after the birth/expulsion) 3
Yes, after the birth 4

If sedatives were administered after diagnosis and/or during the initial stages of labour

36 You have indicated that you were given sedatives after the diagnosis or during the initial stages of labour, which of the following options best describes the reason why they gave you sedatives, at that time?

I asked them to give me something to relax me 1
They told me it would be better if I took something to help me relax 2
They gave me sedatives without consulting with me .. 3

37 Did they explain the effects of the sedatives before they gave them to you?

Yes, they explained it very well 1
Yes, but not fully 2
No 3

If sedatives were administered during the birth

38 You have indicated that you were given sedatives during the labour, either just before or after the birth/expulsion, which of the following options best describes the reason why they gave you sedatives, at that time?

I asked them to give me something to relax me 1
They told me it would be better if I took something to help me relax 2
They gave me sedatives without consulting with me .. 3

39 Did they explain the effects of the sedatives before they gave them to you?

Yes, they explained it very well 1
Yes, but not fully 2
No 3

If sedatives were administered after the birth

40 You have indicated that you were given sedatives after the birth, which of the following options best describes the reason why they gave you sedatives, at that time?

I asked them to give me something to relax me 1
They told me it would be better if I took something to help me relax 2
They gave me sedatives without consulting with me .. 3

41 Did they explain the effects of the sedatives before they gave them to you?

Yes, they explained it very well 1
Yes, but not fully 2
No 3

42 Do you feel that the sedatives affected your memories of the time in the hospital?

Yes, a lot (I feel that I don't remember important things) 1
Yes, a little (but I feel that I remember most important things) 2
I don't think the sedatives affected my memory 3

43 After the birth, did you or your partner see your baby?

No 1
Yes, but not me, only my partner 2
Yes, but only me .... 3 → P44 t P50
Yes, but only my partner .... 4 → P44 t P50

44 Did another family member or friends see the baby/ies?
Tick all that apply

No, no one else 1
Yes, a grandparent or other family member 2
Yes a friend 3

If the mother didn't see the baby…

45 You have indicated that you didn't see your baby. Please choose the number which best reflects your current level of agreement or disagreement with each of the following statements. If you are not sure, use the category 'neither agree nor disagree'. Please only use this category when you definitely don't have a clear opinion.

1 = I completely agree
2 = I agree
3 = I neither agree nor disagree
4 = I disagree
5 = I completely disagree

I received enough information about the decision to see or not see my baby/ies	1	2	3	4	5
They advised me that it would be better to not see my baby/ies	1	2	3	4	5
Even though I decided to not see my baby I felt pressured to do so	1	2	3	4	5
My partner and I had different opinions about seeing the baby/ies	1	2	3	4	5
I regret not seeing my baby/ies	1	2	3	4	5

46 At any time did a health professional tell you that you couldn't see your baby?

No 1
Yes .... 2 → P49

If the respondent was told she couldn't se her baby

47 Please indicate who told you that you couldn't see your baby/ies:
Tick all that apply.

A doctor 1
A midwife or a nurse 2
Other, please specify 3
Open text (short)

48 What reason did they give for saying that you couldn't see your baby/ies?
Please use the space below to tell us

Open text (long)

If the mother didn't see her baby/ies...

49 Is there anything else related to not seeing your baby, which we haven't asked about, that you would like to tell us?
Please use the space below to tell us

Open text (long)

If the mother saw her baby..

50 You indicated that you saw your baby after the birth, where did you see him/her?
Tick all that apply

In the room where I was staying 1
In the emergency room/intensive care unit 2
In the doctor's office/ consulting room 3
In the delivery room 4
In the room just off the delivery room 5
In the family room 6
In the neonatal unit 7
Surgery 8
Surgical reanimation 9
Other please specify 10
Open text (short)

If the mother saw her baby...

51 Please indicate if you or you partner did any of the following when you saw your baby/ies:
Tick all that apply

	Me	Me and my partner	My partner	No
Touch him/her	1	2	3	4
Hold him/her	1	2	3	4
Dress or wrap him/her	1	2	3	4
Wash him/her	1	2	3	4
Spend some time with him or her (vigil)	1	2	3	4
Take photographs	1	2	3	4

If the mother saw her baby...

52 In total how much time, approximately, did you spend with your baby?

1-2 minutes 1
3-5 minutes 2
6-20 minutes 3
21-60 minutes 4
1 to 2 hours 5
More than 2 hours 6
I don't know/ I can't say 7

If the mother saw her baby...

53 You indicated that you saw your baby after the birth. Please choose the number which best reflects your current level of agreement or disagreement with each of the following statements. If you are not sure, use the category 'neither agree nor disagree'. Please only use this category when you definitely don't have a clear opinion.

1 = I completely agree
2 = I agree
3 = I neither agree nor disagree
4 = I disagree
5 = I completely disagree

I received enough information about the decision to see or not see my baby/ies	1	2	3	4	5
They advised me that it would be better to not see my baby/ies	1	2	3	4	5
I felt pressured to see my baby/ies	1	2	3	4	5
My baby was presented to me in a respectful and affectionate manner	1	2	3	4	5
The place where I saw my baby/ies was private	1	2	3	4	5
I felt that I could spend as much time with my baby/ies as I wanted	1	2	3	4	5
The professionals were respectful in their physical treatment of my baby/ies	1	2	3	4	5
The professionals participated in the process of seeing / holding / spending time with my baby/ies	1	2	3	4	5
My partner and I had different opinions about seeing the baby/ies	1	2	3	4	5
It was a good decision to see my baby/ies	1	2	3	4	5

If the mother saw her baby...

54 Is there anything else related to seeing your baby that we haven't asked about and that you would like to tell us?
Please use the space below to tell us

Open text (long)

55 When the midwives or nurses spoke to you about the baby, how did they refer to him/her?
Tick all that apply

By his/her name 1
The baby 2
The fetus 3
Him or her 4
I don't know / I can't remember 5

56 When the doctors spoke to you about the baby, how did they refer to him/her?
Tick all that apply

By his/her name 1
The baby 2
The fetus 3
Him or her 4
I don't know / I can't remember 5

57 Which of the following items did you keep from the hospital?
Tick all that apply

None 1
Photographs 2
Hand and/or foot prints 3
Lock of hair 4
Identification bracelet 5
Ultrasound 6
Clothing, blankets 7
Umbilical cord clamp 8
Medical paperwork 9
Other, please specify 10

Open text (short)

58 Do you have any object or physical memory from the hospital or from during the pregnancy that is especially important to you and your relationship with your baby?
Please use the space below to tell us

Open text (long)

59 Were you asked about religious or spiritual beliefs at any time during the hospital stay?

Yes 1
No 2
I don't know / I can't remember 3

60 Were you asked if you would like to talk to psychologist, trained in intrauterine/neonatal grief, while you were in the hospital?

Yes 1
No .... 2 → P62

If the mother was offered the possibility of seeing a psychologist...

61 Did you accept the offer to see a psychologist?

Yes 1
No 2

62 Now, we'd like you to think about your interactions with the doctors, midwives and nurses.

Please choose the number which best reflects your current level of agreement or disagreement with each of the following statements. If you are not sure, use the category 'neither agree nor disagree'. Please only use this category when you definitely don't have a clear opinion.

1 = I completely agree
2 = I agree
3 = I neither agree nor disagree
4 = I disagree
5 = I completely disagree

I felt that the professionals listened to me	1	2	3	4	5
I felt that I could express myself emotionally in front of the professionals	1	2	3	4	5
The professionals were always respectful to me and my family	1	2	3	4	5
They gave me/ us sufficient information to help make the decisions we had to make	1	2	3	4	5
The professionals were sensitive in their use of language	1	2	3	4	5
Although I lost my baby I was treated like a mother	1	2	3	4	5
I felt emotionally supported by the doctors (e.g. gynaecologists and obstetricians)	1	2	3	4	5
I felt emotionally supported by the nurses and midwives	1	2	3	4	5
I felt I could ask questions if I wanted to	1	2	3	4	5
They helped me/ us to keep physical memories of the baby, such as photographs, identification bracelet, etc.	1	2	3	4	5
Some of the professionals treated me well and others poorly	1	2	3	4	5
The nurses and midwives seemed to know how to deal with cases of pregnancy loss	1	2	3	4	5
The doctors seemed to know how to deal with cases of pregnancy loss	1	2	3	4	5

63 Which of the following pathology studies or tests were you offered (later we'll ask about which studies were conducted)?
Tick all that apply

None 1
General autopsy/ necropsy 2
Autopsy of the placenta 3
Biopsy 4
Phenotypic study (genetic test) 5
Other, please specify 6
Open text (short)

64 Who, if anyone, explained the possibilities of conducting pathology studies or tests?
Tick all that apply

No one 1
A doctor 2
A pathologist 3
A nurse or a midwife 4
Other, please specify 5
Open text (short)

If someone explained the possibility of having pathology studies conducted..

65 Please indicate when you were spoken to about the possibility of having pathology tests conducted:
Tick all that apply

Before the birth 1
During the labour/birth 2
After the birth 3

66 We asked you what pathology tests were offered, now we'd like you to tell us which studies/tests were conducted:
Tick all that apply

General autopsy/ necropsy 1
Autopsy of the placenta .... 2 → P68
Biopsy .... 3 → P68
Phenotypic study (genetic test) .... 4 → P75
None, no pathology study was conducted .... 5 → P75

If a general autopsy was conducted…

67 Did you or your partner sign a consent form for the autopsy?

Yes 1
No 2
Don't know / can't remember 3

If a general autopsy or biopsy was conducted…

68 How long did it take for the autopsy or biopsy results to arrive?
If both an autopsy and biopsy were conducted, answer in relation to the autopsy

Less than 1 month 1
1-3 months 2
4-6 months 3
More than 6 months 4
Still haven't arrived .... 5 → P75

If the results of the autopsy / biopsy have arrived...

69 Which of the following best describes how the results arrived or were delivered?

By post 1
By appointment in the hospital/During a revision 2
By email 3
Going and asking/ making a complaint 4
Other, please specify 5
Open text (short)

If the results of the autopsy / biopsy have arrived...

70 Please indicate who, if anyone, explained the results of the autopsy or biopsy:
Tick all that apply

No one 1
A doctor 2
A pathologist 3
A nurse or a midwife 4
Other, please specify 6
Open text (short)

If the results of the autopsy were explained...

71 With respect to the explanation of the results of he autopsy or biopsy, please choose the number which best reflects your current level of agreement or disagreement with the following statement.

If you are not sure, use the category 'neither agree nor disagree'. Please only use this category when you definitely don't have a clear opinion.

1 = I completely agree
2 = I agree
3 = I neither agree nor disagree
4 = I disagree
5 = I completely disagree

I received a clear and understandable explanation of the results of the autopsy or biopsy............ 1 2 3 4 5

If the results of the autopsy / biopsy have arrived...

72 Did the autopsy or biopsy identify a cause of death?

Yes............ 1
Yes, but not definitive............ 2
No............ 3

If the results of the autopsy (not biopsy) have arrived...

73 Which of the following best describes your current feelings about the decision to authorize the autopsy?

It was a good decision............ 1
I'm undecided, I'm not sure if it was a good decision.... 2
I wish that it hadn't been done............ 3

If a general autopsy or biopsy was conducted…

74 Is there anything in relation to the autopsy or biopsy that we haven't asked that you'd like to tell us about?
Please use the space below

Open text (long)

75 Who explained the procedures and options for the disposition of the body?
Tick all that apply

No one............ 1
A doctor............ 2
A midwife or a nurse............ 3
Someone from the funeral home............ 4
Social worker............ 5
Hospital porter............ 6
Administrator............ 7
Don't know, my partner or other family member took charge of the arrangements for disposition............ 8
Other, please specify............ 9
Open text (short)

76 Which of the following options best describes the funeral procedure or the method of disposition of the body?

The body was donated to research............ 1 → P78
Private burial............ 2 → P78
Private cremation througha funeral home (ashes recovered)............ 3 → P78
Cremation in the hospital (no ashes recovered)............ 4
They didn't return the body to us as it was an early loss............ 5 → P78
They told us tat if there was an autopsy that we couldn't recover the body............ 6 → P78
Burial in a common plot (managed by the hospital)............ 7
Private cremation (no ashes recovered)...... 8
I don't know / no options given............ 9
Other, please specify............ 10 → P78
Open text (short)

77 You have indicated that you chose to have the body cremated in the hospital, without possibility of recovering the ashes, why did you choose that option?
Please use the space below

Open text (long)

78 Now, thinking about the procedures related to care in the hospital, please choose the number which best reflects your current level of agreement or disagreement with each of the following statements.

If you are not sure, use the category 'neither agree nor disagree'. Please only use this category when you definitely don't have a clear opinion.

1 = I completely agree
2 = I agree
3 = I neither agree nor disagree
4 = I disagree
5 = I completely disagree

They explained the birth process in cases of loss in a clearly understandable way........ 1 2 3 4 5
They presented me/us with the official forms and paperwork (e.g. related to the death, autopsy, statistics) at an appropriate time............ 1 2 3 4 5
The room where I was accommodated was peaceful and quiet place............ 1 2 3 4 5
All the personnel on the ward were aware of my situation............ 1 2 3 4 5
In general they kept informed me/us well informed about all the steps and procedures during the hospital stay............ 1 2 3 4 5
The doctors seemed competent in their work............ 1 2 3 4 5
The nurses and midwives seemed competent in their work............ 1 2 3 4 5
The doctors and nurses/midwives seemed to work well as a team............ 1 2 3 4 5
There was one professional who guided me/us through the whole process............ 1 2 3 4 5
I felt that I was in control of the decisions related to medical aspects of care (e.g. the birth, sedatives/medication)............ 1 2 3 4 5
I felt that I was in control of the decisions related to ritual (e.g. seeing/ holding)............ 1 2 3 4 5

79 Please, using the scale below, indicate how much information you received (verbally or in writing) for each of the following areas, during the hospital stay:

1 = None
2 = Little
3 = Quite a bit
4 = A lot

Information about the possibility of keeping physical mementos of the baby, such as photographs, identification bracelet etc..	1	2	3	4
Information about the grief process	1	2	3	4
Information about where to find useful information about perinatal bereavement (e.g. web pages, books)	1	2	3	4
Information about self-care for lactation and after discharge	1	2	3	4
Information about the disposition of the corpse (e.g. the funeral arrangements, cremation etc.)	1	2	3	4
Information about the autopsy and other medical/pathology tests	1	2	3	4

80 Thinking about the hospital stay, what was the thing that helped you most (something someone said or did), if anything?

Open text (long)

81 Thinking about the hospital stay, what was the thing that helped you least (something someone said or did), if anything?

Open text (long)

82 Each of the items is a statement of thoughts and feelings that some people have concerning a loss such as yours. There are no right or wrong responses to these statements.

Choose the number which best reflects your current level of agreement or disagreement with each of the following statements. If you are not sure, use the category 'neither agree nor disagree'. Please only use this category when you definitely don't have a clear opinion.

IMPORTANT: These questions are related to **how you feel now**, not at the time when you suffered the loss.

1 = I completely agree
2 = I agree
3 = I neither agree nor disagree
4 = I disagree
5 = I completely disagree

I feel depressed	1	2	3	4	5
I find it hard to get along with certain people	1	2	3	4	5
I feel empty inside	1	2	3	4	5
I can't keep up with my normal activities	1	2	3	4	5
I feel a need to talk about the baby	1	2	3	4	5
I am grieving for the baby	1	2	3	4	5
I am frightened	1	2	3	4	5
I have considered suicide since the loss	1	2	3	4	5
I take medicine for my nerves	1	2	3	4	5
I very much miss the baby	1	2	3	4	5
I feel I have adjusted well to the loss	1	2	3	4	5
It is painful to recall memories of the loss	1	2	3	4	5
I get upset when I think about the baby	1	2	3	4	5
I cry when I think about him/her	1	2	3	4	5
I feel guilty when I think about the baby	1	2	3	4	5
I feel physically ill when I think about the baby	1	2	3	4	5
I feel unprotected in a dangerous world since he/she died	1	2	3	4	5
I try to laugh, but nothing seems funny anymore	1	2	3	4	5
Time passes so slowly since the baby died	1	2	3	4	5
The best part of me died with the baby	1	2	3	4	5
I have let people down since the baby died	1	2	3	4	5
I feel worthless since he/she died	1	2	3	4	5
I blame myself for the baby's death	1	2	3	4	5
I get cross at my friends and relatives more than I should	1	2	3	4	5
Sometimes I feel like I need a professional counsellor to help me get my life back together again	1	2	3	4	5
I feel as though I'm just existing and not really living since he/she died	1	2	3	4	5
I feel so lonely since he/she died	1	2	3	4	5
I feel somewhat apart and remote, even among friends	1	2	3	4	5
Its safer not to love	1	2	3	4	5
I feel depressed	1	2	3	4	5
I find it hard to get along with certain people	1	2	3	4	5
I feel empty inside	1	2	3	4	5
I can't keep up with my normal activities	1	2	3	4	5

If the death occurred at 22 weeks gestation or later

83 Before you were discharged did they give you (or your partner) the Birth Registration/Statistics Form (the BEP)?

The BEP is a document from the Civil Register, that contains the details of the birth, the mother and father, the gender of the baby, number of weeks gestation, etc. It is a document that the gynaecologist should give you filled in and signed.

No	1
Yes, completely filled in	2
Yes, partially filled in	3
Yes, but it wasn't filled in	4
I don't know / I don't remember	5

If BEP has been received

84 Have you, or another person, taken the BEP to the Civil Registry in your municipality?

Yes, I/we took it to the civil registry	1
Yes, the funeral home took charge	2
I/we still haven't gotten round to it	3
No I/we forgot to do it	4
No, we didn't know that you had to take it to the civil registry	5
I don't know / I don't remember	6

85 After being discharged from the hospital, which of the following options best describes the follow-up care that you had?
Tick all that apply

I haven't had any follow-up since discharge/ not yet because it's too early	1
I went for a check-up in the same hospital	2
I had a house call from the health centre midwife	3
I had an appointment with the midwife in the health centre	4
I had an appointment with the doctor in the health centre	5
I had a follow-up with mental health services	6
I had a follow-up appointment with the gynaecologist at 6 weeks	7
I had a follow-up appointment with the gynaecologist 1-3 weeks after discharge	8
I had a check-up with a private gynaecologist	9
I had follow-up appointments with specialist diagnostics	10
I had to emergencies because of complications	11
I had continued periodic check-ups	12
Other, please specify	13

Open text (short)

86 Thinking about how you feel at present, which of the following options best describes how you are coping with the death of your baby?

Very bad (every day seems bad) 1
Quite bad (more bad days than good days) 2
Neither good nor bad (good days and bad days about the same) 3
Quite good (more good days than bad days) 4
Very good (most days are good, but maybe with the odd bad one) 5
I don't know/ can't say 6

87 Have you received any psychological help, support or therapy at any of the following times?
Tick all that apply

No 1
After being discharged from the hospital 2
During a subsequent pregnancy 3
Both after discharge and during a subsequent pregnancy 4

88 Where did you go for psychological support or therapy?
Tick all that apply

Psychologist / psychiatrist public mental health service 1
Nurse from the public mental health service 2
Private psychologist / psychiatrist - non specialist in grief 3
Private psychologist / psychiatrist - specialist in grief . 4
Private psychologist / psychiatrist - specialist in perinatal grief 5
General grief support group 6
Perinatal grief support group 7
Alternative/natural therapy 8
Psychologist / psychiatrist public mental health service - specialist in perinatal grief 9
Other, please specify 10
Open text (short)

If the respondent is still married/ civil union/ cohabiting with partner

89 Has your partner received any psychological help, support or therapy at any of the following times?
Tick all that apply

No 1
After being discharged from the hospital 1
During a subsequent pregnancy 2
Both after discharge and during a subsequent pregnancy 4

90 Where did your partner go for psychological support or therapy?
Tick all that apply

Psychologist / psychiatrist public mental health service 1
Nurse from the public mental health service ....
Private psychologist / psychiatrist - non specialist in grief 2
Private psychologist / psychiatrist - specialist in grief ... 3
Private psychologist / psychiatrist - specialist in perinatal grief 4
General grief support group 6
Perinatal grief support group 7
Alternative/natural therapy 8
Psychologist / psychiatrist public mental health service - specialist in perinatal grief 9
Other, please specify 10
Open text (short)

If the pregnancy was singleton

91 If the possibility had existed would you have donated your breast milk to a bank?

Yes, definitely 1
Yes, probably 2
I don't know, I'm not sure 3
No, definitely not 4

92 Please indicate if you thought there was any medical negligence in your case?

No 1
Yes, we made a claim 2
Yes, but we didn't make a claim/ report it 3
I don't know, I'm not sure 4

93 Have you ever looked for information on perinatal death on the Internet?
Tick all that apply

No, never 1
Yes, during the hospital stay 2
Yes, after being discharged 3

94 Have you had any contact with other parents or a support group/association that helps parents who have experienced a perinatal death?
Tick all that apply

No 1
Yes, I've been in contact with a support association by telephone or email 2
Yes, I've participated in an online support group or forum 3
Yes, I've attended or participated in a support group/ self-help group 4
Yes, I've been in contact with other mothers/fathers, but not in a formal support group 5
Yes, I've attended a commemoration event 6

95 How would you rate the level of support that you have received from the following people?

No support = 1
A little support= 2
Quite a bit of support = 3
A lot of support = 4
Not relevant (don't know any such person) = 5

1) Nurses and/or midwives	1	2	3	4	5
2) Doctors	1	2	3	4	5
3) Your partner/husband	1	2	3	4	5
4) Close family	1	2	3	4	5
5) Family in general	1	2	3	4	5
6) Close friends	1	2	3	4	5
7) Friends in general	1	2	3	4	5
8) A psychologist/psychiatrist	1	2	3	4	5
9) Other mothers/fathers who have experienced a perinatal death	1	2	3	4	5
10) An online support group	1	2	3	4	5
11) A support group/ self-help group (physically attend)	1	2	3	4	5
12) Work colleagues	1	2	3	4	5
13) A support association	1	2	3	4	5

96 Thinking about how you feel now about the care that you received in the hospital choose the number which best reflects your current level of agreement or disagreement with each of the following two statements.

If you are not sure, use the category 'neither agree nor disagree'. Please only use this category when you definitely don't have a clear opinion.

1 = I completely agree
2 = I agree
3 = I neither agree nor disagree
4 = I disagree
5 = I completely disagree

Despite the circumstances I feel satisfied with the care that I received in the hospital 1 2 3 4 5

I would recommend this hospital to other parents 1 2 3 4 5

97 Which of the following best defines your religious beliefs?

Catholic 1
Evangelic or protestant 2
Jewish 3
Muslim 4
Mormon 5
Orthodox 6
Buddhist 7
Jehovah's Witness 8
Non-believer / agnostic 9
Atheist 10
At the moment I have strong doubts about my religious beliefs 11
Other 12

If the respondent is married/ civil union/ cohabiting with partner

98 Which of the following best describes your partner's educational level?

5 or less years of schooling 1
5 or more years of schooling but without completing the lower second level exams 2
Lower second level 3
Upper second level 4
Professional/vocational level I 5
Professional/vocational level II 6
Third level diploma 7
Third level degree 8
Master or doctorate 9

If the respondent is married/ civil union/ cohabiting with partner

99 Which of the following best describes your partner's occupation?

Professional or technical 1
Executive/director of a public body or a company 2
Administrative worker 3
Merchant or salesperson 4
Service sector worker 5
Agricultural or fisheries sector worker 6
Production worker, transport equipment drivers and labourers (non-agricultural) 7
Member of the armed forces 8
Student 9
Home worker 10
Pensioner, retired or in receipt of an annuity 11
Person who can't be classified 12
Professional or technical 13

100 Which of the following social classes would you say you belong to?

High 1
Middle to high 2
Middle 3
Middle to lower 4
Lower 5

101 Which of the following best describes where you live?

In the provincial capital 1
In the suburbs of the provincial capital 2
In a small city or large town 3
In a small town 4
In a village 5
On a farm or in a house in the countryside 6

102 Currently, between all members of the family and including all income sources, which of the following categories best describes your family's total monthly income?

Less than or equal to 300 € *1*
Between 301 and 600 € *2*
Between 601 and 900 € *3*
Between 901 and 1.200 € *4*
Between 1.201 and 1.800 € *5*
Between 1.801 and 2.400 € *6*
Between 2.401 and 3.000 € *7*
Between 3.000 and 4.500 € *8*
Between 4.501 and 6.000 € *9*
More than 6.000 € *10*

103 You've reached the end of the questionnaire! Would you be interested in participating in other research projects?

Yes, I would like to participate 1
Maybe, but I'd like to receive more information before committing 2
No, thank you .... 3 → P105

104 You have indicated that you would like to participate in other research studies, please leave your name, telephone number and email address and we will be in contact.

Please remember that your personal details are strictly confidential and will never appear in relation to the research.

Name	
Telephone number	
E-mail	

If the respondent answered that they don't wish to participate in further research...

105 Would you like to receive information about the results of the research?

Yes 1
No .... 3 → P107

If the respondent has indicated that they would like to received information about the results of the research...

106 You have indicated that you would like to receive information about the results of the research, please leave your name, telephone number and email address and we will be in contact.

Please remember that your personal details are strictly confidential and will never appear in relation to the research.

Name	
E-mail	

107 If there is anything about the survey that you would like to tell us, something that we have forgotten to ask or a suggestion for improvement, please use the space below.

Open text (long)

Thank you very much for all your help, we promise to use the information that you have provided to promote improvements in the care that parents receive after a perinatal death.

HELP US TO CONTACT OTHER MOTHERS

It's very important for the research that we contact as many mothers as possible. If you know a mother or family that have experienced a loss your pregnancy after week 16 we would be very grateful if you could copy the link below and send it to them by email or communicate it by other means.

Link to send the survey to other mothers:

xxxxx

INFORMATION ABOUT THE SURVEY:

If you have any doubts or questions about the research, please get in touch with Paul Cassidy by email: investigación@umamanita.es

INFORMATION ABOUT PERINATAL GRIEF AND SUPPORT

If you would like to receive more information about perinatal grief or support, please visit Umamanta's webpage or facebook page:

http://www.umamanita.es
https://www.facebook.com/uma.manita?fref=ts

For information about group support, click here:

Group support Facebook page

We can also recommend the following associations that also support mothers and fathers after pregnancy loss and perinatal death:

Petits am llum
http://www.petitsambllum.org/

Superando un aborto (SUA)
http://superandounaborto.foroactivo.com/
