# Supplementary material for: Care quality following intrauterine death in Spanish hospitals: results from an online survey
Source: BMC Pregnancy Childbirth. 2018 Jan 10;18:22. doi: 10.1186/s12884-017-1630-z (PMC5763533; doi:10.1186/s12884-017-1630-z)
Supplement: Supplementary file 3 — Additional details on the study methodology. (PDF 172 kb) [file 12884_2017_1630_MOESM3_ESM.pdf]

ADDITIONAL FILE 1.
ADDITIONAL DETAILS ON THE STUDY METHODOLOGY

ARTICLE TITLE
Care quality following intrauterine death in Spanish hospitals: Results from an online survey

AUTHOR
Paul Richard Cassidy BBS MPhil

Doctoral student
Universidad Complutense de Madrid
Facultad de Ciencia Política y Sociología
Somosaguas, Pozuelo de Alarcon
Madrid, 28223
Email: pcassidy@ucm.es

Researcher
Umamanita (Stillbirth Charity)
C/ Hierbabuena 15, Esc B, 4 Izq
Madrid, 28039
Email: paulcassidy@umamanita.es

1.1. BACKGROUND

The "Perinatal Bereavement Care Project" is a long-term study initiated by the support association Umamanita, which has been designed and managed in a voluntary and fully independent capacity by Paul Richard Cassidy, BBS MPhil, as part of a masters and doctoral research programme at the University Complutense of Madrid and Umamanita's (stillbirth charity) research programme. The primary objective of the research was to establish data on healthcare practices in Spanish hospitals after perinatal bereavement and to elicit women's views on the quality of care that they received. The project represents the first ever attempt to gather quantitative data on perinatal bereavement care at a national level within the Spanish healthcare system.

At a specific level the project set out with a number of sub-objectives:

1. To establish measures of care quality after perinatal bereavement in relation to objective aspects of care, such as the rate of postmortem contact, mode of delivery, types of pathology studies conducted, etc.
2. To establish measures of care quality related to women's perceptions and attitudes towards the care that they received, including such aspects of care as the diagnosis/death notification, interactions with health professionals (emotional support, communication) and procedural aspects of care (coordination of care, competence, information, arrangements for disposition).
3. To develop and psychometrically validate a scale to measure perceived care quality in hospitals following perinatal bereavement and to establish the aspects of care that most significantly influence patient satisfaction (related to objective 2).
4. To establish what factors related to healthcare, if any, influence women's decisions to have post-mortem contact after intrauterine death.
5. To carry out a transcultural adaptation and psychometric validation of the Perinatal Grief Scale (Poitvin et al., 1989) to Spanish.
6. To establish levels and sources of social support post discharge.

The project has been undertaken on a completely voluntary basis, without subsidy or funding.

1.2. STUDY POPULATION

The study population was defined as women who had experienced an intrauterine death (stillbirths or termination of pregnancy for medical reasons) ≥16 weeks gestation up to an including intrapartum deaths.

Although perinatal deaths are typically defined as occurring from 20-24 weeks onwards, the most commonly used definition defines from as occurring from 22 weeks gestation or 28 weeks for purposes of measuring mortality rates [1]. The reason for including deaths/losses from 16 weeks onwards (otherwise known as late miscarriages) relates to the fact that clinical definitions of perinatal death relate to the potential viability of the fetus in the case of premature birth and have little relevance to parents' experiences from the perspective of grief and loss. Although some studies have found a relationship between gestational age and grief intensity [2–6], many others have found the opposite [7–12]. Regardless, all studies find a high degree of variability between cases, which at the very least makes gestational age a poor predictor of grief and not a useful variable for guiding care practices and interactions with women and their partners. However, this is not to say that gestational age doesn't affect clinical treatment (mode of delivery for example) or the reality of hospital care. Following consultations with parents' support associations (in particular one association focused on support after miscarriage) and pilot testing it was decided to set the lower bound of gestational age at 16 weeks. This represented an expansion of the original intention to interview cases ≥20 weeks gestation. Neonatal deaths were not included in the study for two reasons: firstly, it would have necessitated an overly long and complex questionnaire to study both stillbirth care practices and neonatal care practices in detail; and secondly, it was felt that neonatal bereavement care was far more advanced than stillbirth care, though that is difficult to substantiate.

In terms of the type of death included, the study encompasses both stillbirths and terminations of pregnancy for medical reasons (fetal anomaly, threat to the mother's life or selective reduction in multiple gestation pregnancies). Terminations of pregnancy were included because research evidence shows that grief experiences are very similar to stillbirth [13–17]. It was also felt that, other than the clinical aspect of the diagnosis, care practices are also very similar [18-20]. Additionally, there was a concern that the experiences and needs of parents who go through a termination are often doubly stigmatised and misunderstood, so the promoting organisation and participants in the consultation felt that they should be included.

Women's partners were not invited to participate in the study. This was a difficult decision but the primary objective of the study was to benchmark care quality, if partners had been included in the study, duplication of cases (i.e. the death/loss) was a very real possibility, with no way to differentiate. Additionally, the way that the questionnaire was designed wasn't suitable for a partners experience because of the way it tracks care procedures from diagnosis to discharge/follow-up. It may have been possible to incorporate the partner's experience but it would have necessitated, in effect, the development of two distinct instruments, which was beyond the resource means of the project. In the questions where we include data on partners (e.g. seeing/holding) it is based on the mother's testimony.

1.3. INSTRUMENT DEVELOPMENT

The instrument was developed over a period of 18 months and through a number of stages:

Literature review

An extensive review of the literature was conducted in the following theme areas: perinatal bereavement (journal articles and guidelines published by support associations and professional bodies); perinatal bereavement care (journal articles and published guidelines on care effectiveness, care quality and care satisfaction); literature on scale/construct development and measurement in the area of service quality and patient satisfaction and care quality; literature on designing measurement scales; and literature on designing online questionnaires.

Exploratory interviews and participant observation

A series of exploratory interviews and consultations were carried out with one of the founders of the perinatal bereavement support association Umamanita. Subsequently, two couples (both stillbirth cases) were interviewed in their homes and one mother was interviewed by Skype (selective reduction of a multiple pregnancy for medical reasons). Exploratory interviews were also conducted with health professionals (two midwives and one hospital based psychiatrist). The researcher also attended support association planning meetings and a number of training courses for health professionals, provided by the support association Umamanita.

Draft questionnaire phase

The first draft questionnaire was developed with feedback from the support association Umamanita.

The Spanish (Castellano) version of the Perinatal Grief Scale [21] was developed on the basis of two Latin Spanish versions [22,23] through an adaptation process, lead by the researcher José Manuel Romero Sanchez in conjunction with Juan Carlos Paramio Cuevas.

Content validation phase

A panel of experts was created for the second phase of development, which included 14 mothers/fathers who had loss a child through perinatal death and 10 health professionals with

experience in perinatal bereavement care (nurses, midwives, neonatologists and psychologists/psychiatrists[1]). Each participant received two documents, firstly, a copy of the draft questionnaire, which they had to revise, comment on and propose changes. Secondly, they received a copy of the draft care quality scale containing a battery of 54 proposed measurement items, which they had to rank individually in terms of the importance of each item, as well as making suggestions to eliminate or add new items.

Prior to this phase the second general draft version of the questionnaire was developed and sent to each of the experts for final comments.

Cognitive interviews

Once the content validation phase had been completed, three recorded (and transcribed) cognitive interviews (lasting from 1 to 1.5 hours) were conducted with two couples and one mother, during which the interviewees discussed each question, clarifying meaning and raising any doubts or possible misunderstandings.

Pilot interviews

The online version of the questionnaire was piloted in 3 steps: Firstly, 3 mothers carried out a general test run. Following minor modifications, 18 pilot interviews were conducted, with extra text boxes added at the end of each section so that the interviewees could leave comments. Following final changes, 3 further pilots were conducted before launch. Pilot interviews were not included in the final dataset.

Questionnaire structure

The questionnaire contains 102 questions, arranged in the following sections and thematic areas:

- Introductory socio-demographic questions
- Details of the loss/death
- Pregnancy history (previous pregnancies, previous losses, complications during the pregnancy)
- Diagnosis and death notification
- Administration of sedatives/tranquilizers
- Details related to the birth (mode of delivery, accompaniment)
- Post-mortem contact and linking objects
- Perceived care quality scale to measure interactions and relations with health professionals (diagnosis, emotional support, control of decision-making, coordination of care and procedures)
- Details related to the autopsy and procedures for the disposition of the body/remains
- Availability of psychological support during the hospital stay
- Spanish version of the Perinatal Grief Scale (33 item scale)
- Post discharge follow-up and contact with mental health services and support associations
- Scale for the measurement of social support post-discharge
- Final socio-demographic and economic data
- Contact information for support associations

Digital version of the questionnaire

The digital version of the questionnaire was developed using Limesurvey, which permitted access control through respondents' IP addresses, meaning that once a questionnaire had been completed from a particular computer the survey couldn't be accessed again from the same terminal, unless the

[1] Despite attempts no gynaecologist/obstetrician participated

IP was reset. Respondents had the possibility of saving their responses and returning later, if they were unable to complete the questionnaire in one sitting.

The online version of the questionnaire also permitted randomised ordering of items in battery type questions to avoid presentation and fatigue related bias.

The paper version of the questionnaire in Spanish and a translated English version are available in Additional files 2 and 3.

1.4. PARTICIPANT SAFETY

The introductory page of the survey highlighted the study's overall objective, identified the promoter (Umamanita) and specified the basic criteria for participant inclusion. In addition, potential participants were informed that some of the questions might be emotionally sensitive and were assured that in accordance with Spanish data protection law that the information they provided would be treated as strictly confidential. Participants were provided with a contact email where they could make contact with the principal researcher if they had any doubts about the questionnaire or methodology.

The survey was anonymous, respondents were not required to leave any contact details, but had the option to do so at the end of the questionnaire if they wanted to receive information about the research results. This option was included because of feedback during the questionnaire development and pilot testing where there was a strong feeling was expressed that follow up with survey results would be important to ensure respondents that their participant and time would have some material benefit for other families going through the same experience in the future, one of the primary reasons for participating. Before leaving their details, the respondents were assured (again) that their personal details would never be published in relation to the data and that they were covered by the Spanish data protection act, whose conditions specify that the data be stored on a password protected computer. This data is uniquely available to the principal researcher (Paul Cassidy). In total 86% of respondents left their contact details (first name and email).

At the very end of the questionnaire the respondents were provided with the contact details of various parents' support associations in case they felt the need to contact them for support.

1.5. FIELDWORK

The fieldwork took place over a period of three years, from the 24th June 2013 to the 29th June 2016.

Subjects were recruited through the snowballing technique (non-probabilistic convenience sampling) using the following methods and reference chains:

- Publicity through the support association websites, facebook pages and contact lists, such as Umamanita, Petits Amb Llum and the online forum Superando un Aborto.
- Contact with self-help support groups associated with these support associations.
- Writing to blogs and web pages dedicated to perinatal bereavement support, asking for coverage of the survey.
- Writing to Umamanita's list of health professionals, which includes: obstetricians, gynaecologists, nurses, midwives, to ask them to inform patients.
- Writing to Umamanita's list of mental health professionals, including: psychologists, psychiatrists, and mental health nurses, to ask them to inform patients.
- Writing to professional associations of midwives (regional) and obstetricians/gynaecologists, seeking coverage on social media.
- Publication of a press release, which achieved some coverage in national and regional press, such as: eleconomista.es, portalesmedicos.com, lainformación.com, alicanteactualidad.com, iberoamerica.net and elmundodigital.es.

1.6. RESPONSES AND DATA PURIFICATION

During the fieldwork period, a total of 1,082 surveys were completed. However, following data purification, the number of valid cases was reduced to 929, of which 796 had occurred within the 5 years previous to the completion of the survey.

Table 1, below, details the data purification process that identified 52 neonatal cases, 29 cases where the death had not taken place within Spanish territory (Latin American countries), 24 duplicate cases and 2 cases where the birth had occurred at home. 36 cases were eliminated due to the questionnaire not being sufficiently completed (missing fields count), containing inconsistent data, having been completed too rapidly, or a combination of all three.

Identification of neonatal cases

The introductory text clearly specified that neonatal cases should not complete the questionnaire. As such, the large degree of participation of neonatal cases was a surprise, given the length and detail of the questionnaire. This may point to the fact that many parents wish to tell their story and that this is an under researched area. Neonatal cases were identified through a number of methods: firstly 'neonatal' was left as a possible response option to the question on type of death. Secondly, in the question on "where" the parents saw their baby the open-text response option "other" captured answers such as "NICU". Finally, other open-ended questions captured responses that helped to identify these cases. It should also be noted that if a perinatal death occurs during labour or very shortly beforehand the baby may be put on an artificial ventilation system and moved to the NICU. Exploratory qualitative interviews, conducted for this study, have found that this opens up the question that if a baby was not breathing when born and subsequently placed on a ventilation system, when precisely did the death occur? Was it in fact a stillbirth, intrapartum or neonatal death? In this sense, it may be that some of the cases classified as neonatal deaths were in fact stillbirth or intrapartum deaths. Additionally, some late terminations of pregnancy or terminations beyond 19-20 weeks, where feticide was not carried out, may have resulted in a live birth [24-25].

Table 1. Data purification

	**Time between loss/death and completion of the questionnaire**		
	**≤60 months** *n* (%)	**>60 months** *n* (%)	**Total** *n* (%)
Valid cases	796 (86.0)	133 (85.3)	929 (85.9)
Non-valid cases - external to the study population			
Neonatal deaths in Spain	45 (4.9)	7 (4.5)	52 (4.8)
Cases external to Spanish national health system*	25 (2.7)	4 (2,6)	29 (2.7)
Participant hadn't yet given birth	3 (0.3)	2 (1.3)	5 (0.5)
Cases with no hospital based care (home births)	2 (0.2)	0 (0.0)	2 (0,2)
Father, partner or other family member	1 (0.1)	0 (0.0)	1 (0.1)
Non-valid cases - poor data quality or duplication			
Too many missing data fields	27 (2.9)	9 (5.8)	36 (3.3)
Excess of time between starting and completing the questionnaire (2 weeks)	2 (0.2)	0 (0.0)	2 (0.2)
Duplicated cases**	24 (2.6)	1 (0.6)	25 (2.3)
Total	926 (100.0)	156 (100.0)	1082 (100.0)

**All Spanish speaking Latin American countries*

Identification of cases external to the Spanish national territory

Cases outside the national territory of Spain were principally identified through the response option that asked for the hospital's name, open-ended responses (some cases volunteered the information at the end of the questionnaire) and the choice of "province" in the geographic location question (e.g. many Mexican respondents choose "Guadalajara", also a Spanish province, but one with a small population).

Elimination of cases with an excess of missing fields

A count of missing fields was conducted on key data fields in various sections of the questionnaire (pregnancy history, socio-demographic data, data related to the pregnancy/death, objective data and subjective data). Based on a total count of missing data and a case-by-case analysis respondents were included or excluded. In a survey of this length (>400 possible variables/data fields) a certain level of missing data is to be expected. In the final data set, 39.6% of cases had no missing data and 43.1% had 1-3 missing data fields. In 4 cases the missing data count is over 10, but these cases were admitted because the lost data relates to specific questions with a large number of variables, such as a scale, whereby the rest of the data set was well completed and the data judged to be valuable.

Duplicated cases

A number of duplicated cases (24), where the same respondent had completed the questionnaire on 2 occasions were identified. These cases were identified by comparing cases and also by analysing contact details left at the end of the questionnaire (as mentioned above 86% of respondents left their contact details in order to receive the survey results). Why might this have happened? Firstly, as the fieldwork took place over a period of three years with a number of rounds of publicity, it may be that the call to participate was misinterpreted, that the respondent forgot having completed the questionnaire or they were unsure that their response had been properly recorded. In theory the computer IP address control system built into the Limesurvey software should have controlled this possibility, but it can only be effective if the respondent uses the same hardware device. It is a concern from a methodological perspective and strategies to counter act it should be put into place, such as warnings to respondents that the survey is going through multiple rounds of recruitment. In this instance having respondents email data proved particularly useful for identifying duplications.

1.7. ABANDONED INTERVIEWS

Rate of abandonment

In total 632 interviews were initiated and abandoned before completion. 59.2% of incomplete interviews were abandoned before 5 minutes and 75.2% before 10 minutes (table 2).

Given the emotionally sensitive nature of the survey, and the fact that the questionnaire is particularly long and detailed, a higher rate of abandonment was expected. In reality, the rate of abandonment was not excessively high, particularly if those not reaching 1 minute or 5 minutes are excluded.

Table 2. Analysis of abandoned interviews (duration of the abandoned interview)

	n	(%)
< 1 minute	201	(31.8)
1 a <2 minutes	59	(41.1)
2 a <5 minutes	114	(59.2)
5 a <10 minutes	101	(75.2)
10 a <15 minutes	40	(81.5)
15 a <20 minutes	35	(87.0)
20 a <25 minutes	19	(90.0)
25 a <30 minutes	20	(93.2)
≥30 minutes	43	(100.0)
Total	632	(100.0)

Profile of abandoned interviews

It is important to note that an analysis of the abandoned interviews reveals that their profile (where the data was available from the initial questions) is more or less similar to the final sample, except for one criteria: the number of weeks gestation at the time of death/loss. Of the 632 interviews abandoned, 293 got as far as Question 12 (number of weeks gestation at which the death occurred) and an analysis of these cases shows that they were significantly associated with earlier losses (16 and 19 weeks gestation), compared to the complete sample. This difference is indicative that the original design of the questionnaire was for perinatal deaths from 20 weeks onwards and that the decision to widen the population to include earlier losses came about during the content validation phase of the questionnaire and after the first phase of pilot interviews. It is evident that the questionnaire is not perfectly designed for these early losses, particularly at 16 and 17 weeks, given that they fall outside of any biological definition of perinatal death and that there is greater variation in the way that the baby might be lost, for example, some might suffer a haemorrhage at home and many don't spend the night in the hospital. Consequently, for some cases before 20 weeks gestation the questionnaire might be difficult to complete or not correspond exactly to their experience. However, inclusion of this cohort was justified by the fact that 128 women who had experienced a loss before 20 weeks gestation did complete the questionnaire, allowing a very valuable comparison of experiences before and after 20 weeks gestation. That said, in terms of the introductory text and filter questions, the questionnaire could have been improved to help reduce the rate of abandonment of these women.

1.9. CODING

Note on the coding options in the postmortem question:

In the question related to autopsy and postmortem examination, a term “necropsy” was erroneously included in a response option distinct to “autopsy”, which may have lead to a small amount of confusion for respondents. However, the vast majority of respondents chose the response option termed “general autopsy” and an analysis of open-ended response options permitted the identification of 36 additional cases where an autopsy/necropsy had been conducted. Following this adjustment, the final autopsy rate for the entire sample increased from 56.8% to 61.4%. This final autopsy rate is very close to that found in national data for stillbirth cases ≥20 weeks gestation in the same period (2010-2015) covered by the study: 70.5% in this study vs. 71.2% in the national data [26].

1.8. ANALYSIS

Note on the analytic categories related to gestational age

While formal biological definitions of perinatal death tend to make distinctions on the basis of gestational age before and after 22/28 weeks (early and late stillbirths) the analysis established that 26 weeks gestation is a better predictor of changes in care in the Spanish healthcare system. This is probably related to Spanish legislation (Law of the 15th of June, 1957) that makes legal distinctions before and after 180 days gestation (26 weeks), which has come to affect hospital protocols in relation to the disposition of the body/remains and postmortem examination.

Effect size value for contingency tables

Table 3 sets out the effect size (Phi and Cràmer's V) interpretation criteria used to evaluate the contingency table analysis.

Table 3. Interpretation criteria for effect size (Phi and Cràmer's V) for contingency tables

	Effect size		
**Degrees of freedom(*df*)**	**Small**	**Medium**	**Large**
1	.10	.30	.50
2	.07	.21	.35
3	.06	.17	.29
4	.05	.15	.25
5	.04	.13	.22

Source: Cohen (1988)/ Gravetter & Wallanu (2014) [27,28]

REFERENCES

1. World Health Organisation. Neonatal and Perinatal Mortality: Country, Regional and Global Estimates. Geneva: World Health Organisation; 2006.
2. Benfield DG, Leib SA, Vollman JH. Grief response of parents to neonatal death and parent participation in deciding care. Pediatrics. 1978;62(2):171-178.
3. Peppers LG, Knapp RJ. Maternal reactions to involuntary fetal/infant death. Psychiatry. 1980;43(May):155-159.
4. Clarke-Smith A, Borgers SB. Parental grief response. Omega J Death Dying. 1988;19(3):203-214.
5. Zeanah CH, Danis B, Hirshberg L, Dietz L. Initial adaptation in mothers and fathers following perinatal loss. Infant Ment Health J. 1995;16(2):80-93. http://doi.org/10.1002/1097-0355.
6. Rich DE. The impact of postpregnancy loss services on grief outcome: integrating research and practice in the design of perinatal bereavement programs. Illn Cris Loss. 2000;8(3):244-264. http://doi.org/10.1177/105413730000800303.
7. Kirkley-Best E. Grief in response to prenatal loss: an argument for the earliest maternal attachment. Doctoral Thesis. 1981.

8. Dyregrov A, Matthiesen SB. Anxiety and vulnerability in parents following the death of an infant. Scand J Psychol. 1987;28:16-25.
9. Theut SK, Pedersen FA, Zaslow MJ, Cain RL, Rabinovich BA, Morihisa JM. Perinatal loss and parental bereavement. Am J Psychiatry. 1989;146(5):635-639. http://doi.org/10.1176/ajp.146.5.635.
10. Lasker JN, Toedter LJ. Acute versus chronic grief: the case of pregnancy loss. Am J Orthopsychiatry. 1991;61(4):510-522. http://doi.org/10.1037/h0079288.
11. Goldbach KRC, Dunn DS, Toedter LJ, Lasker JN. The effects of gestacional age, gender on grief after pregnancy loss. Am J Orthopsychiatry. 1991;61(3):461-467. http://doi.org/10.1037/h0079261.
12. Cuisinier MCJ, Kuijpers JC, Hoogduin CAL, de Graauw CPHM, Janssen HJEM. Miscarriage and stillbirth: time since the loss, grief intensity and satisfaction with care. Eur J Obstet Gynecol Reprod Biol. 1993;30(163-168). http://doi.org/10.1016/0028-2243(93)90066-L.
13. Kersting A, Reutemann M, Ohrmann P, et al. Grief after termination of pregnancy due to fetal malformation. 2004;(June):163-169. http://doi.org/10.1080/01674820400002162.
14. Kersting A, Dorsch M, Kreulich C, et al. Trauma and grief 2 – 7 years after termination of pregnancy because of fetal anomalies – a pilot study. J Psychosom Obstet Gynecol. 2005;26(1):9-14. http://doi.org/10.1080/01443610400022967.
15. Hunt K, France E, Ziebland S, Field K, Wyke S. "My brain couldn"t move from planning a birth to planning a funeral': a qualitative study of parents' experiences of decisions after ending a pregnancy for fetal abnormality. Int J Nurs Stud. 2009;46(8):1111-1121. http://doi.org/10.1016/j.ijnurstu.2008.12.004.
16. France EF, Hunt K, Zieband S, Wyke S. What parents say about disclosing the end of their pregnancy due to fetal abnormality. Midwifery. 2013;29(1):24-32. http://doi.org/10.1016/j.midw.2011.10.006.
17. Korenromp MJ, Page-Christiaens GC, van den Bout J, Mulder EJ, Visser GHA. Adjustment to termination of pregnancy for fetal anomaly: a longitudinal study in women at 4, 8, and 16 months. Am J Obstet Gynecol. 2009;201:160.e1-e7. http://doi.org/10.1016/j.ajog.2009.04.007.
18. Royal College of Obstetricians & Gynaecologists. Termination of Pregnancy for Fetal Abnormality in England, Scotland and Wales. Report of a Working Party. London: Royal College of Obstetricians; 2010.
19. Asplin N, Wessel H, Marions L, Georgsson-Öhman S. Pregnancy termination due to fetal anomaly: women's reactions, satisfaction and experiences of care. Midwifery. 2014;30(6):620-627. http://doi.org/10.1016/j.midw.2013.10.013.
20. Geerinck-Vercammen C, Kanhai H. Coping with termination of pregnancy for fetal abnormality in a supportive environment. Prenat Diagn. 2003;23(7):543-548. http://doi.org/10.1002/pd.636.
21. Potvin L, Lasker JN, Toedter LJ. Measuring grief: a short version of the Perinatal Grief Scale. J Psychopathol Behav Assess. 1989;11(1):29-45. http://doi.org/10.1007/BF00962697.
22. Capitulo KL, Ramirez M, Grigoroff-Aponte B, Vahey D c. Psychometric testing of the new Spanish short version of the Perinatal Grief Scale to measure perinatal grief in Spanish-speaking parents. Hisp Heal Care Int. 2010;8(3):125-136. http://doi.org/10.1891/1540-4153.8.3.125.
23. Mota-González C, Calleja-Bello N, Aldana-Calva E, Gómez-López ME, Sánchez-Pichardo MA. Escala de duelo perinatal: validación en mujeres mexicanas con pérdida gestacional. Rev Latinoam Psicol. 2011;43(3):419-428. http://doi.org/10.14349/rlp.v43i3.854.
24. Wyldes MP, Tonks AM. Termination of pregnancy for fetal anomaly: a population-based study 1995 to 2004. BJOG An Int J Obstet Gynaecol. 2007;114(5):639-642. http://doi.org/10.1111/j.1471-0528.2007.01279.x.
25. Movva VC, Fleener D, Rijhsinghani A. Unintended live births following second trimester pregnancy termination by labor induction. J Reprod Med. 2016;61(7-8):357-360.

26. Instituto Nacional de Estadísticas. Demografía y población, Estadísticas de nacimiento, Muertes Fetales (Microdatos). 2010–2015. http://www.ine.es
27. Cohen J. Statistical Power Analysis for the Behavioral Sciences. Revised Ed. New York: Academic Free Press; 1977.
28. Gravetter FJ, Wallnau LB. Essentials of Statistics for the Behavioural Sciences. 8th ed. Belmont, CA: Wadsworth, Cengage Learning; 2014.
